# Emergent structure and dynamics of tropical forest-grassland landscapes

**DOI:** 10.1073/pnas.2211853120

**Published:** 2023-10-30

**Authors:** Bert Wuyts, Jan Sieber

**Affiliations:** ^a^Centre for Systems, Dynamics and Control, Department of Mathematics and Statistics, University of Exeter, EX4 4QF, United Kingdom

**Keywords:** alternative stable states, tropical forest, cellular automata, mean field, nonlinear dynamics

## Abstract

Previous work indicates that tropical forest can exist as an alternative stable state to savanna. Therefore, perturbation by climate change or human impact may lead to crossing of a tipping point beyond which there is rapid forest dieback that is not easily reversed. A hypothesized mechanism for such bistability is feedback between fire and vegetation, where fire spreads as a contagion process on grass patches. Theoretical models have largely implemented this mechanism implicitly, by assuming a threshold dependence of fire spread on flammable vegetation. Here, we show how the nonlinear dynamics and bistability emerge spontaneously, without assuming equations or thresholds for fire spread. We find that the forest geometry causes the nonlinearity that induces bistability. We demonstrate this in three steps. First, we model forest and fire as interacting contagion processes on grass patches, showing that spatial structure emerges due to two counteracting effects on the forest perimeter: forest expansion by dispersal and forest erosion by fires originating in adjacent grassland. Then, we derive a landscape-scale balance equation in which these two effects link forest geometry and dynamics: Forest expands proportionally to its perimeter, while it shrinks proportionally to its perimeter weighted by adjacent grassland area. Finally, we show that these perimeter quantities introduce nonlinearity in our balance equation and lead to bistability. Relying on the link between structure and dynamics, we propose a forest resilience indicator that could be used for targeted conservation or restoration.

## Significance

Tropical forests may face the risk of abrupt dieback due to amplifying feedback between forest loss and fire spread. Considering the patch-scale rules of forest and fire spread, we find that forest expands into grassland at a rate proportional to its perimeter, while it recedes at a rate proportional to its perimeter and the area of adjacent grassland. Looking at the landscape-scale balance of changes in forest area, we find that these two quantities respectively appear in the gain and loss parts of the equation. Such a relation between spatial structure and expected change of forest area can help identify which parts of the landscape are best targeted for conservation or restoration to avert forest dieback.

Satellite (1, 2) and ground observations (3, 4) show that tropical forest (high tree cover) and tropical savanna (low tree cover) can exist under the same environmental conditions, making the distribution of tree cover bimodal. On the one hand, fire exclusion experiments have shown that fire can maintain low tree cover (5). On the other hand, fire occurs almost exclusively below a tree cover threshold of about 40% (1, 6–9), which is consistent with fire being a contagion process on grass patches (10, 11), while tree patches block fire. Such a highly nonlinear response of fire to grass together with an empirically consistent response of vegetation to fire was shown to be sufficient for inducing bistability in simple models (12). Taken together, the bimodality, the mutual interaction between fire and vegetation, and the availability of a plausible underlying mechanism suggests that tropical forest and savanna are alternative stable states, maintained by feedback between vegetation and fire (1), and between which transitions would neither be gradual nor easily reversed (13, 14).

Bistability of forest and savanna has been studied with a variety of modeling approaches, which can be classified as microscopic versus mean-field models. The underlying processes concern the spatiotemporal population dynamics of discrete vegetation patches, which can spread or block fire. These can be most realistically modeled by microscopic models, such as interacting particle systems (15) or cellular automata (16), which consider the stochastic dynamics of such discrete constituents interacting in a spatial domain according to simple rules. However, as microscopic models are hard to analyze, one usually looks for a coarse-grained approximation that permits analysis. Mean-field models provide such an approximation, typically in the form of a small number of differential equations that describe the average properties of the considered populations through time, such as cover fractions of each species. If the averages are taken over the whole landscape, the resulting mean-field model is nonspatial and describes macroscopic dynamics via ordinary differential equations (ODEs) (9, 12, 17). If averages are taken over a neighborhood, the mean-field model is spatial and describes the dynamics on a mesoscopic scale, via partial differential (18, 19), spatial difference (20); spatial mean field in ref. 21], or partial integro-differential equations (mean field in ref. 15). Mean-field models owe their simple closed form to an assumption of statistical independence between species’ occurrences in space (e.g., refs. 22 and 23), which permits writing the interaction between any two species as the product of their occurrences. However, assuming statistical independence in space implies neglect of spatial structure.

Despite their disregard for spatial structure and resulting biases (e.g., refs. 22 and 24), mean-field models have been indispensable tools for gaining theoretical insight into alternative stable tree cover states in the tropics. The Staver–Levin model of tropical tree cover bistability (12) is a nonspatial mean-field model in which the variables represent grass and tree cover fractions in the landscape, with interaction between species captured as the product of their cover fractions. Fire spread is not included explicitly. Instead, the effects of fire on vegetation are implicitly accounted for by making the relevant conversion rates a threshold function of grass cover, where the threshold corresponds to the point where large contiguous grass patches emerge, also known as the percolation threshold (25, 26). The Staver–Levin model has provided a first proof of principle for alternative stable tree cover states in the tropics, and showed additional complex behaviors, such as cycles and stochastic resonance (12, 17). Spatial mean-field models of the Staver–Levin model further showed emergent phenomena due to spatial interaction on mesoscopic scales, such as traveling and pinning fronts between states (8, 18, 19, 21); spatial mean field in ref. 15], front curvature effects (19, 21), pattern formation (27), and coexistence states (28). Even though they are spatial, they are still mean-field models, as they do not consider the fundamental spreading processes of forest and fire on patches, but use equations, with implicit assumptions on the spatial structure of the patches at finer scales than those modeled.

The effect of this fine-grained spatial structure can only be studied via microscopic models. Schertzer et al. (10) proposed a cellular automaton implementation of the Staver–Levin model in which the effect of fire is still captured implicitly, as a threshold function of flammable vegetation. The form of this vegetation-fire relation was obtained from separate simulations of fire spread as a standard percolation process. The cellular automaton and its mean-field approximation were shown to exhibit bistability. Thereby, Schertzer et al. (10) provided the first mechanistic explanation of the role of fire as a percolation process in bistability of tropical tree cover. It also justifies the qualitative form of the fire-vegetation dependence assumed in mean-field models. The more recent interacting particle system by Patterson et al. (15) follows a similar approach, by implementing fire as a threshold function of neighborhood grass cover, where the threshold is assumed to match with that of site percolation (25, 26). However, standard percolation theory assumes that the occurrences of spreading cells at different points in space are statistically independent (section 1.1 in ref. 25). Thus, if fire spread is approximated as a standard percolation process, one disregards the spatial structure of flammable vegetation. Hence, although the microscopic Staver–Levin models (10, 15) consider the fine-grained patch structure, they still rely on a mean-field assumption in their implicit treatment of fire, making them prone to biases in regimes with spatial structure. To avoid these biases, microscopic models require explicit consideration of fire spread in interaction with the vegetation landscape, such as in the cellular automaton by Hébert-Dufresne et al. (16) (see also ref. 29). In this cellular automaton, forest bistability emerges only from simple microscopic rules of vegetation and fire spread, i.e., without assuming equations or thresholds for the effects of fire. Note that larger-scale forest transitions have also been modeled with a cellular automaton, with the effects of climate and fire as spatially heterogeneous parameters (30).

In this work, we examine the spontaneous emergence of nonlinear dynamics and bistability of tropical forest from the patch-scale rules of forest and fire spread. We first use the cellular automaton of Hébert-Dufresne et al. (16) to observe the emergent structure and bistability in simulations. Next, based on the observations that forest and fire spread occur near the forest perimeter and on separated timescales, we set up a macroscopic balance equation of forest area change (Eq. **9**). This enables us to analyze the emergent dynamics as a function of the relevant structure and will show that the nonlinearity is caused by the forest geometry. Then, we derive a forest resilience indicator based on our balance equation, providing a proposed link between the geometry and resilience of tropical forest. Finally, we compare our results against mean-field approximations. This will show that the assumption of the absence of spatial correlations is strongly violated, particularly near the tipping threshold of forest dieback, while mean-field models still permit accurate expressions for the spatially uncorrelated regime.

## Results

### The FGBA Probabilistic Cellular Automaton.

**Fig. 1.**
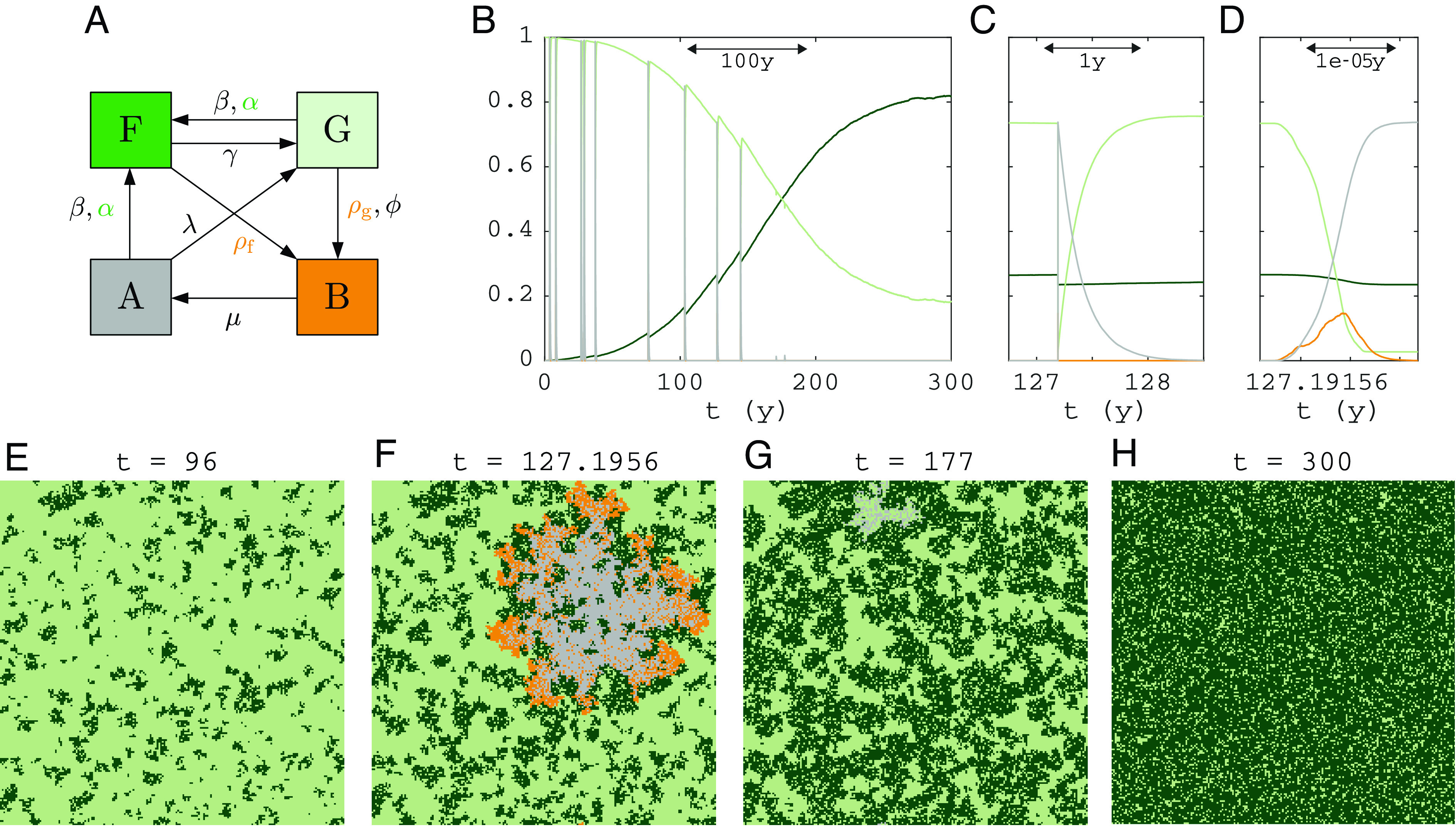
The FGBA stochastic cellular automaton: (*A*) state transition diagram (colored rates: spread to neighboring cell, black rates: spontaneous conversion within cell), (*B*) example time series of a simulation starting at zero tree cover, (*C* and *D*) $10^{2}\times$ and $10^{7}\times$ zoom of (*B*), (*E*–*H*) snapshots of a simulation at indicated times for low fire ignition rate (ϕN=0.075). The fire in (*F*) spreads throughout grassland in the whole domain whereas that in (*G*) went extinct locally because forest splits grassland in clusters (notice the area of ash near the top). Remaining parameters are shown in Table 1. Domain size: 200 × 200 cells.

The FGBA probabilistic cellular automaton (adapted from ref. 16—see Fig. 1 and *Materials and Methods*) models the stochastic dynamics of tropical vegetation and fire on a square lattice and in continuous time. The key empirical facts of tropical forest and fire dynamics captured by the FGBA automaton are the following: i) fires only naturally ignite in grasslands but they can spread into forest, ii) fires spread more easily in grassland than in forest, such that forests suppress fires, albeit imperfectly, iii) forest dynamics occur on a strongly separated timescale from fire spread and grass regrowth.

This results in the following reaction rules in the cellular automaton. At any time, each lattice cell can be in one of four states: F—forest, G—grass, B—burning, and A—ash. Conversions between these states can occur spontaneously or due to spread to neighboring cells (Fig. 1*A* and Table 1). The spontaneous conversions are as follows: forest recruitment on grass or ash cells due to long-distance seed dispersal or from a homogeneous seed bank (G→F or A→F at rate β), forest mortality (F→G at rate γ), fire ignition on grass cells (G→B at rate ϕ), and grass regrowth on ash cells (A→G at rate λ). The conversions due to spread to neighbors are as follows: forest recruitment due to short-distance seed dispersal on grass or ash cells (GF→FF,AF→FF at rate α), fire spread on grass (GB→BB at rate $\rho_{g}$) or on tree cells (FB→BB at rate $\rho_{f}$). Chosen parameters are in the ranges empirically justified by ref. 16 for a square domain of 100 × 100 cells, with cell size Δx=Δy=30 m. The timescale separation between fire and forest dynamics implies that the rates satisfy $\rho_{g},\rho_{f},\mu ,\lambda \gg \alpha ,\beta ,\gamma$. In particular, we choose

[1] $$\rho_{\text{g}},\mu ∼10^{6}>\rho_{\text{f}}∼10^{5}\gg 1{\text{y}}^{-1},$$

[2] $$\alpha ,\beta ,\gamma ∼10^{\left[-4,-2\right]}\ll \lambda ∼1{\text{y}}^{-1}.$$

**Table 1.** Reaction rules and rates (y${}^{-1}$)

	Spontaneous		Spread	
	$\;\text{G},\;\text{A}\overset{\beta }{\to }\;\text{F},$	$\beta =2\times 10^{-4}$	$\;\text{GF},\;\text{AF}\overset{\alpha }{\to }\;\text{FF},$	$\alpha_{\;}=3\times 10^{-2}$
	$\;\text{F}\overset{\gamma }{\to }\;\text{G},$	$\gamma =2\times 10^{-2}$		
	$\;\text{G}\overset{\phi }{\to }\;\text{B},$	$\phi =\left[0,2\right]\times 10^{-4}$	$\;\text{GB}\overset{\rho_{\;}\text{g}}{\to }\;\text{BB},$	$\rho_{\;}\text{g}=9\times 10^{6}$
	$\;\text{B}\overset{\mu }{\to }\;\text{A},$	$\mu =10^{6}$	$\;\text{FB}\overset{\rho_{\;}\text{f}}{\to }\;\text{BB},$	$\rho_{\;}\text{f}=1.11\times 10^{5}$
	$\;\text{A}\overset{\lambda }{\to }\;\text{G},$	λ=5		

So, fire spreading and extinction $\rho_{g}$, $\rho_{f}$, μ occur on the scale of hours, while grass regrows on ash over months (λ) and forest spread, growth, and mortality α, β, γ occur over decades. We take fire ignition rate ϕ∼1/N such that fires spontaneously occur about once per year in the modeled area. Fig. 1*B*–*D* shows a time profile for fractions of cells in each state during a simulation with low fire ignition rate ϕ, starting from an all-grass state. Due to the low fire ignition rate, the only stable steady state is a nearly closed canopy (reached after 300 y, Fig. 1*H*). Before canopy closure, brief events of rapidly spreading fire counteract a gradual spread of forest. After canopy closure, fires are unable to spread. Timescale separation of forest dynamics (Fig. 1*B*), grass regrowth (Fig. 1*C*), and fire spread (Fig. 1*D*) shows clearly.

**Fig. 2.**
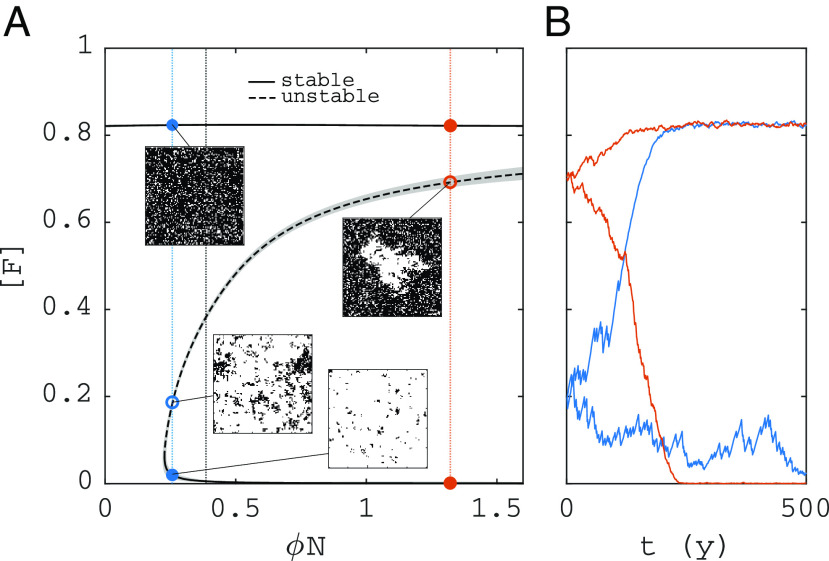
Steady states and bistability of forest area in the FGBA cellular automaton. (*A*) Bifurcation diagram of forest area fraction [F] versus fire ignition rate ϕ (shade: two-standard deviation confidence interval of the mean). (*B*) Simulations initiated at two different points on the saddle (ϕN=0.257 and ϕN=1.32). Remaining parameters are shown in Table 1. Domain size: 100 × 100 cells.

Fig. 2*A* shows a bifurcation diagram of steady-state forest area in the FGBA cellular automaton, denoted by [F] (Eq. **18**), versus fire ignition rate ϕ. Unstable steady states (saddles) were obtained by applying feedback control to the simulations (*Materials and Methods*). Bistability occurs above a critical ignition rate ϕ, with alternative stable states grassland ([F]≈0) and forest ([F]≈0.83). Simulations initiated at the saddle will tip randomly up or down (Fig. 2*B*). Near the lower end of the bistability range, the saddle solution is fairly homogeneous, but for higher ϕ values, a single hole of grass in forest arises (*Insets* in Fig. 2*A*).

### Fast and Slow Subprocesses.

The timescale separation (Eqs. **1** and **2**) permits treatment of the joint vegetation and fire dynamics as a fast–slow system. Fire spread occurs on the fast timescale, where the vegetation landscape is treated as constant. Forest dynamics occur on the slow timescale, where the effects of fire are a steady-state function of the vegetation landscape.

### Fast Process: Fires Spreading in a Given Landscape.

On the timescale of a single fire event, forest dynamics are negligible (α,β,γ≪1/d) such that we can consider the total landscape of forest patches as fixed. For each ignition event, this results in the following dynamics. A fire ignites on a grass cell, and then spreads across its grassland cluster at a rate $\rho_{g}$ per BG pair, after which it reaches the interface with adjacent forest, where it starts intruding the forest at a rate $\rho_{f}$ per BF pair. At any time, a burning cell can stop burning spontaneously, converting to ash at a rate μ. The probabilities of fire spreading into a neighboring grass or forest cell before the originating cell stops burning are given by

[3] $$p_{g}:=\frac{\rho_{g}}{\rho_{g}+\mu }=0.9,\;p_{f}:=\frac{\rho_{f}}{\rho_{f}+\mu }=0.1,$$

where we have shown the chosen values in our simulations (adopted from ref. 16). Since regrowth of grass and ignition of new fires occur at a much slower rate than fire spread ($\phi N≲\lambda \ll \rho_{f},\mu ,\rho_{g}$) and our domain is relatively small (*SI Appendix*, section S2B), we observe repeated fire spreading events well separated in time (Fig. 1*B*), each ending with spontaneous extinction.

When a fire in grassland cluster with index j reaches its interface with adjacent forest, the resulting forest loss due to this single fire event can be approximated as (*Materials and Methods*):

[4] $$\Delta_{F,j}^{\mathrm{loss}}:=p_{f}{\left[\mathrm{FG}\right]}_{j},$$

where ${\left[\mathrm{FG}\right]}_{j}$ counts the number of forest cells adjacent to grassland cluster j (with both sides of the equation optionally normalized by N). This approximation relies on the assumptions that the fire reaches the whole interface with forest (i.e., $p_{g}\to 1$) and only once per fire (i.e., $\rho_{g}\gg \lambda \gg \phi N$), and that $p_{f}$ is small.

### Slow Processes: Forest Demography and Fire Damage.

Forest demography and loss due to repeated fires occur on the slow timescale. Writing the number of forest-grass neighbor pairs as [FG] (divided by N, equivalently the total perimeter of forest or grass patches, see Eq. **18**), the dynamics for tree recruitment and mortality result in an expected rate of change for [F]:

[5] $$\Delta_{F}^{\mathrm{gain}}:=\beta \left[G\right]-\gamma \left[F\right]+\alpha \left[\mathrm{FG}\right].$$

In Eq. **5**, the rates of change are β[G] for spontaneous forest growth on grass, γ[F] for spontaneous forest mortality, and α[FG] for spread of forest into grass at its perimeter.

The rate of forest erosion at its perimeter due to fire damage over many fire events is the weighted sum over all grass clusters $j\;=\;1,...,n_{c}$, i.e.,

[6] $${\text{Δ}}_{\text{F}}^{\text{loss}}:=\sum_{j=1}^{n_{\text{c}}}\phi N{\left[\text{G}\right]}_{j}{\text{Δ}}_{\text{F},j}^{\text{loss}}=\phi Np_{\text{f}}\sum_{j=1}^{n_{\text{c}}}{\left[\text{G}\right]}_{j}{\left[\text{FG}\right]}_{j},$$

where ${\left[G\right]}_{j}$ is the fraction of G cells in grass cluster j (so, $\left[G\right]\;=\;\sum_{j=1}^{n_{c}}{\left[G\right]}_{j}$), $\phi N{\left[G\right]}_{j}$ is the rate at which fires spontaneously ignite in grass cluster j (ϕ is the rate per cell and $N{\left[G\right]}_{j}$ is the area of the cluster), and $\Delta_{F,j}^{\mathrm{loss}}\;=\;p_{f}{\left[\mathrm{FG}\right]}_{j}$ is the conversion of forest to ash caused by each fire event (Eq. **4**) (note also that $\left[\mathrm{FG}\right]\;=\;\sum_{j=1}^{n_{c}}{\left[\mathrm{FG}\right]}_{j}$). By defining the grassland-weighted forest perimeter as

[7] $${\langle [\;\text{FG}]\rangle }_{\text{cg}}:=\sum_{j}^{n_{c}}\frac{{\left[G\right]}_{j}}{\left[G\right]}{\left[\mathrm{FG}\right]}_{j},$$

the expression for forest loss becomes

[8] $$\Delta_{F}^{\mathrm{loss}}=\phi \;p_{f}\;N\left[G\right]{\langle [\;\text{FG}]\rangle }_{\text{cg}}.$$

The grassland-weighted forest perimeter ${\langle [\;\text{FG}]\rangle }_{\text{cg}}$ is the average perimeter of forest clusters weighted by the relative size of their adjacent grass cluster.

### Emergent Slow Dynamics.

We now form the balance between the slow processes discussed above, assuming fire converts trees immediately to grass (i.e., λ≫ϕN). The resulting expected rate of forest area change during a short time interval is

[9] $$\begin{array}{cc} \langle \frac{d[F]}{dt}\rangle = & \Delta_{F}^{\mathrm{gain}}-\Delta_{F}^{\mathrm{loss}},\\ \frac{d[F]}{dt}= & \beta \left[G\right]-\gamma \left[F\right]+\alpha \left[\mathrm{FG}\right]-\phi p_{f}N\left[G\right]{\langle [\;\text{FG}]\rangle }_{\text{cg}}, \end{array}$$

where we used Eqs. **5** and **8**, and assumed on the left-hand side that N is sufficiently large, such that, via the law of large numbers, ⟨d[F]/dt⟩≈d[F]/dt. Eq. **9** can be understood intuitively as forest and grass competing for space within clusters (spontaneous terms) and at their interface (interaction terms). A larger interface [FG] leads simultaneously to faster forest spread (proportional to its perimeter [FG]) and to increased exposure to fires (proportional to its grassland-weighted perimeter ${\langle [\;\text{FG}]\rangle }_{\text{cg}}$). Fires are most damaging to forest when [G] forms a single cluster, i.e., ${\langle [\;\text{FG}]\rangle }_{\text{cg}}\;=\;\left[\mathrm{FG}\right]$, such that each fire reaches the whole interface. Conversely, when forest patches break [G] into several clusters ${\langle [\text{FG}]\rangle }_{\text{cg}}$ is smaller than [FG], such that several ignitions are required to have the same effect, slowing forest erosion down. Additionally, the total amount of grass N[G] determines the number of ignitions and hence the rate at which grass spreads into forest. The parameters determine the relative weight of each of the discussed effects.

**Fig. 3.**
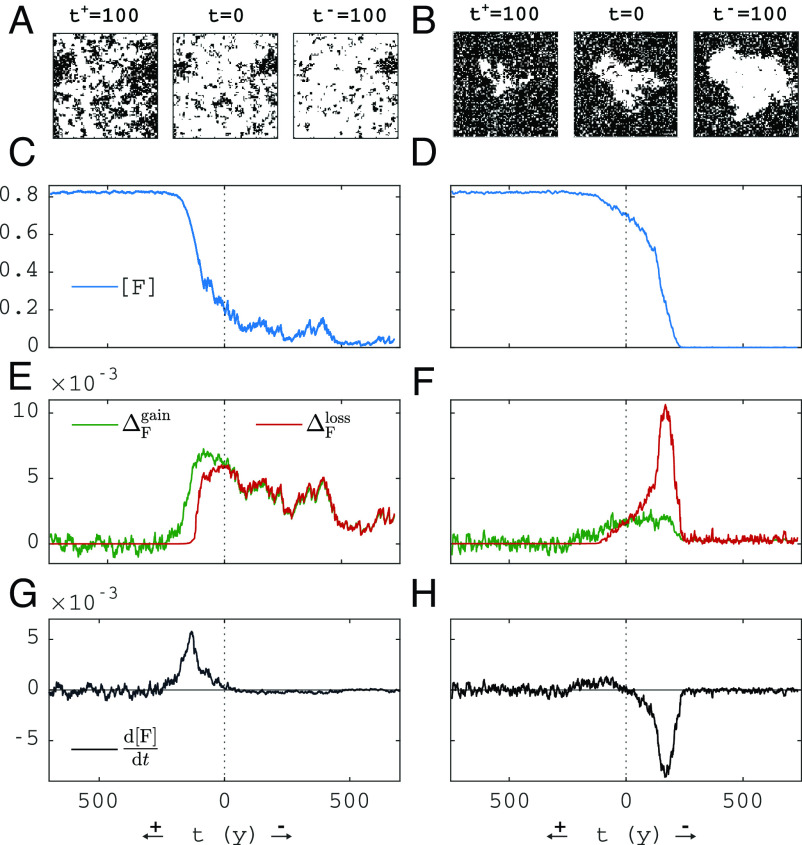
Rate of change according to Eq. **9**. (*A* and *B*) Spatial snapshots at indicated times, (*C* and *D*) time series of [F], (*E* and *F*) time series of $\Delta_{F}^{\mathrm{gain}}$, $\Delta_{F}^{\mathrm{loss}}$, (*G* and *H*) time series of the right-hand side of Eq. **9** (gain minus loss). At t=0, the simulation is started on the saddle (on the left, [F](0)≈0.2 and on the right, [F](0)≈0.7, see blue and red circles in Fig. 2). Toward the left (along $t^{+}$), a simulation that tips up and toward the right (along $t^{-}$) a simulation that tips down is shown. Parameters are shown in Table 1. Columns correspond to leftmost and rightmost vertical dashed lines in Fig. 2 (ϕN=0.257 and ϕN=1.32). Domain size: 100 × 100 cells.

Fig. 3 shows example simulations along trajectories starting from the saddle equilibria of Fig. 2, showing forest area [F] in space and time (*A*–*D*), the gain/loss terms $\Delta_{F}^{\mathrm{gain}}$ and $\Delta_{F}^{\mathrm{loss}}$ defined in Eqs. **5** and **8** (*E* and *F*), and the right-hand side of Eq. **9** (gain minus loss, *G* and *H*). The left column of Fig. 3 shows simulations for low fire ignition rate ϕ and low [F](0), and the right column for high ϕ and high [F](0). Each column shows two realizations, both starting from the same saddle steady state. One realization evolves toward high forest cover, shown on axis $t^{+}$ (increasing to the left from t=0), the other realization evolves toward low forest cover, shown on axis $t^{-}$ (increasing to the right from t=0). In the stable steady states, gain (green) and loss (red) terms vary around the same mean. On the saddle (at t=0), gain and loss functions cross, indicating that the steady states and changes are accurately captured by Eq. **9**. The largest changes in forest cover [F] occur when there are large changes in forest loss due to fire. The snapshots in Fig. 3 *A* and *B* show that the high-cover state changes as an expanding/contracting hole in the forest, whereas the low-cover state is more homogeneous.

### Emergent Nonlinear Relations.

**Fig. 4.**
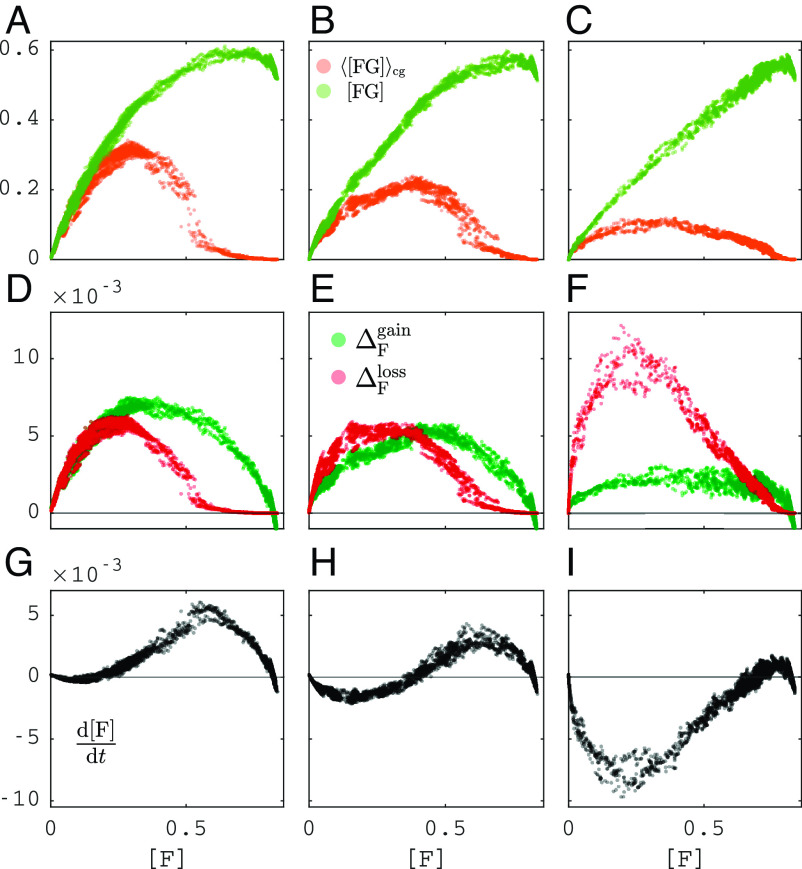
Emergent relations between perimeter quantities and forest area [F]: (*A*–*C*) forest perimeter [FG] and grassland-weighted forest perimeter ${\langle [\text{FG}]\rangle }_{\text{cg}}$, (*D*–*F*) forest gain terms and loss terms in Eqs. **5** and **8**, (*G*–*I*) forest area rate of change (d/dt)[F] from Eq. **9**. Columns correspond to vertical dashed lines in Fig. 2 (ϕN=0.257,ϕN=0.38,ϕN=1.32). Domain size: 100 × 100 cells.

Eq. **9** explains how the rate of change of [F] depends on the perimeter quantities [FG] and ${\langle [\text{FG}]\rangle }_{\text{cg}}$. Fig. 4*A*–*C* shows a scatterplot of [FG](t) and ${\langle [\text{FG}]\rangle }_{\text{cg}}\left(t\right)$ versus [F](t) for three different values of ϕ, and for an ensemble of simulations starting from the saddle in Fig. 2*A*, with each point being a value observed at a discrete observation time. Remarkably, we observe that [FG] and ${\langle [\text{FG}]\rangle }_{\text{cg}}$ lie on a narrow band around some steady-state functions ${\left[\mathrm{FG}\right]}^{*}$ and ${\langle [\text{FG}]\rangle }_{\text{cg}}^{*}$ of [F] (and ϕ), which implies that $\left[\mathrm{FG}\right],{\langle [\text{FG}]\rangle }_{\text{cg}}$ are changing on a much faster timescale, making them slaved to [F]. Fig. 4*D*–*F* shows the terms on the right-hand side of Eq. **9** depending on [F], splitting between gain and loss terms $\Delta_{F}^{\mathrm{gain}},\Delta_{F}^{\mathrm{loss}}$, as defined in Eqs. **5** and **8**. Steady states occur when gain equals loss ($\Delta_{F}^{\mathrm{gain}}\;=\;\Delta_{F}^{\mathrm{loss}}$). The resulting plot of d[F]/dt versus [F] in Fig. 4*G*–*I* clearly shows the bistability of [F].

Replacing the quantities [FG] and ${\langle [\text{FG}]\rangle }_{\text{cg}}$ by their steady-state functions ${\left[\mathrm{FG}\right]}^{*}$ and ${\langle [\text{FG}]\rangle }_{\text{cg}}^{*}$ results in the single-variable ODE for [F],

[10] $$\frac{d[F]}{dt}=\beta \left[G\right]-\gamma \left[F\right]+\alpha {\left[\mathrm{FG}\right]}^{*}-\phi p_{f}N\left[G\right]{\langle [\text{FG}]\rangle }_{\text{cg}}^{*},$$

where ${\left[\mathrm{FG}\right]}^{*},{\langle [\text{FG}]\rangle }_{\text{cg}}^{*}$ are functions of [F] and ϕ (as shown in Fig. 4*A*–*C*), and [G]=1−[F]. With these functions ${\left[\mathrm{FG}\right]}^{*}$ and ${\langle [\text{FG}]\rangle }_{\text{cg}}^{*}$, the observed bistability is caused by a classic double-well potential of the gradient system Eq. **10**. In this ODE, nonlinearities emerge due to the equilibrium dependence of the interface on forest area (affecting ${\left[\mathrm{FG}\right]}^{*}$ and ${\langle [\text{FG}]\rangle }_{\text{cg}}^{*}$), due to the segmentation of grass cells near and below the percolation threshold (affecting ${\langle [\text{FG}]\rangle }_{\text{cg}}^{*}$) and due to dependence of the ignition rate on grass patch size (multiplying ${\langle [\text{FG}]\rangle }_{\text{cg}}^{*}$ with [G]). In *SI Appendix*, Fig. S1, we show the roots of Eq. **10** using a nonparametric fit of ${\left[\mathrm{FG}\right]}^{*}\left(\left[F\right];\phi \right)$ and ${\langle [\text{FG}]\rangle }_{\text{cg}}^{*}\left(\left[F\right];\phi \right)$. These match well with the steady states obtained via control (dot-dashed red).

If there is only one connected component of grass cells, we have ${\langle [\text{FG}]\rangle }_{\text{cg}}\;=\;\left[\mathrm{FG}\right]$, such that Eq. **10** simplifies to

[11] $$\frac{d[F]}{dt}=\beta \left[G\right]-\gamma \left[F\right]+\left(\alpha -\phi p_{f}N\left[G\right]\right){\left[\mathrm{FG}\right]}^{*}.$$

For homogeneous initial conditions (i.e., [F] is about the same in different large subsections of the domain), this approximation is expected to be valid for small [F], where most grass cells belong to the giant connected component. *SI Appendix*, Fig. S1 shows the resulting steady states of Eq. **11** as a function of ϕ and [F] when only using the fit of ${\left[\mathrm{FG}\right]}^{*}\left(\left[F\right];\phi \right)$ (dashed blue). The approximation is good for landscapes with low forest cover ([F]≲0.2). Above [F]≈0.2, it fails because the grassland breaks up into multiple clusters and fires are smaller than in case of a single cluster. Fig. 4*A*–*C* already indicated that the single-cluster approximation is accurate for low forest cover since ${\langle [\text{FG}]\rangle }_{\text{cg}}\;\approx \;\left[\mathrm{FG}\right]$ for low [F] in the scatterplots.

### Resilience to Perturbations.

One can evaluate the right-hand side of Eq. **9** for a landscape before and after application of a small perturbation, to determine whether the perturbation will be dampened or amplified under the dynamics. More precisely, we may define the sensitivity as

[12] $$\lambda_{F}\left(X,\delta X\right):=\frac{\Delta \dot{\left[F\right]}\left(X,\delta X\right)}{\Delta [F](X,\delta X)}=\frac{\dot{\left[F\right]}\left(X+\delta X\right)-\dot{\left[F\right]}\left(X\right)}{\left[F](X+\delta X)-[F](X\right)},$$

for a landscape X and a perturbation δX, where $\dot{\left[F\right]}$ is the right-hand side of Eq. **9**. Negative values of $\lambda_{F}$ correspond to dampening (negative feedback) and positive values to amplification (positive feedback). Given that the dynamics of Eq. **9** are an approximate function of [F] only, the average of $\lambda_{F}\left(X\;+\;\delta X\right)$ over naturally expected perturbations δX (call this ${\overset{¯}{\lambda }}_{F}\left(X\right)$; see *Materials and Methods* and Eq. **26**) can be interpreted as the approximate derivative $d\dot{\left[F\right]}\left(\left[F\right]\right)/d\left[F\right]$, which fully characterizes the local stability of the landscape. Therefore, the sign and magnitude of ${\overset{¯}{\lambda }}_{F}\left(X\right)$ are indicators for the stability or criticality of a landscape. The dependence on only [F] also implies that Eq. **9** is a gradient system, such that ${\overset{¯}{\lambda }}_{F}\left(X\right)$ is the concavity of the potential energy function at X, corresponding to the classic potential-well metaphor of local resilience (14, 31).

**Fig. 5.**
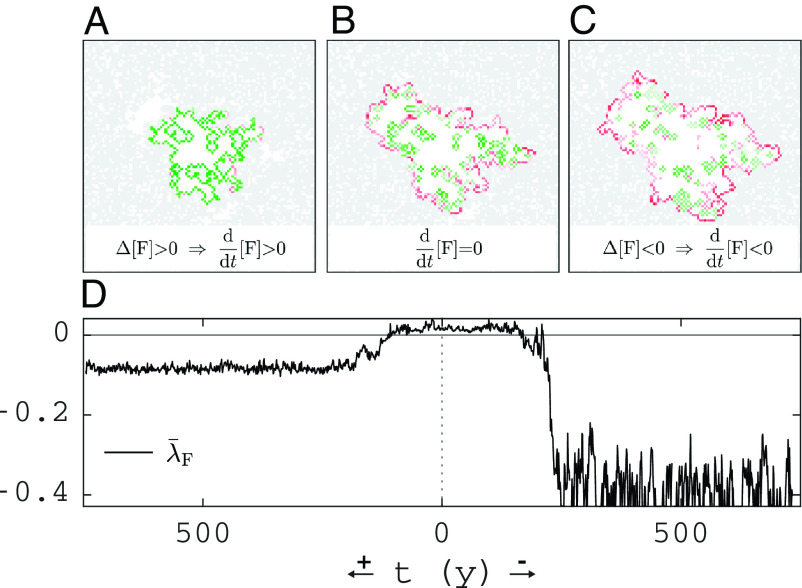
Resilience of forest-grass landscape to perturbations. (*A*–*C*) Spatially resolved contributions to forest gain (green) and loss (red) rates at the forest perimeter of the largest grass cluster: (*B*) for the saddle solution (where $\Delta_{F}^{\mathrm{gain}}\;\approx \;\Delta_{F}^{\mathrm{loss}}$), (*A*) for a perturbation of the saddle with more forest at the perimeter (resulting in $\Delta_{F}^{\mathrm{gain}}\;>\;\Delta_{F}^{\mathrm{loss}}$), and (*C*) for a perturbation of the saddle with less forest at the perimeter (resulting in $\Delta_{F}^{\mathrm{loss}}\;>\;\Delta_{F}^{\mathrm{gain}}$). (*D*) Sensitivity to perturbations (Eq. **12**) for all the traversed landscapes when tipping from the saddle: down to the grassland state ($t^{-}$), or, up to the forest state ($t^{+}$). The used landscapes correspond to the red vertical dashed line in Fig. 2 and the rightmost columns in Figs. 3 and 4.

Fig. 5*D* shows ${\overset{¯}{\lambda }}_{F}\left(X\right)$ for the traversed landscapes when tipping up to the forest or down to the grassland state (same landscapes as in Fig. 3*B*, *D*, *F*, and *G*). Comparison of the magnitude of ${\overset{¯}{\lambda }}_{F}\left(X\right)$ in the alternative stable states reveals that (for parameters of Fig. 3*D*, *F*, and *G*) the grassland state is more resilient than the forest state. Fig. 5*A*–*C* shows the positive feedback for a forest landscape with a hole of critical size (same landscape as shown at t=0 in Fig. 3*B*). Panel *B* shows which cells along the perimeter of the largest grass cluster contribute to the loss term $\Delta_{F}^{\mathrm{loss}}$ (red) and the gain term $\Delta_{F}^{\mathrm{gain}}$ (green). Panel *A* shows the effect of the perturbation obtained by converting the green cells to forest, which causes an increase in $\dot{\left[F\right]}$ (more green in panel *A*). Panel *C* shows the effect of the perturbation converting the red cells to grass, which causes a decrease in $\dot{\left[F\right]}$ (more red in panel *C*), illustrating the spatial distribution of the positive feedback.

One could, in principle, also test the effect of large perturbations and whether they will induce a transition to an alternative stable state, but it is not, in general, clear which perturbations are to be expected. However, in the special case of β=γ=0, when the large perturbation is a single hole in contiguous forest, only the size of the hole matters, and a simple expression for the critical size required to tip abruptly to a nonforest state can be derived (*Materials and Methods* and Eq. **25**).

### Comparison to Mean-field Approximations.

Our analysis of Eq. **9** enabled us to obtain macroscopic steady states and dynamics without making mean-field assumptions. *SI Appendix*, section S3 derives a hierarchy of mean-field models for which we compare their predictions to our results to examine their validity. The simple mean field (*SI Appendix*, section S3) is unable to capture repeated fire extinction on a fast timescale and nearest-neighbor spreading of forest and fire, leading to severe bias. When instead assuming timescale separation between forest and fire dynamics and treating fire as a site percolation process in landscapes with uniform random (i.e., spatially uncorrelated) placement of forest, we obtain the mean-field approximation of Eq. **9**:

[13] $$\frac{\text{d}}{\text{d}t}\left[\text{F}\right]=\beta \left[\text{G}\right]+4\alpha \left[\text{F}\right]\left[\text{G}\right]-\gamma \left[\text{F}\right]-\phi Np_{\text{f}}[\text{G}]{\langle \left[\text{FG}\right]\rangle }_{\text{cg}}^{\text{u}}.$$

In Eq. **13**, we substituted the a-priori unknown forest perimeter [FG] and the grassland-weighted perimeter ${\langle [\text{FG}]\rangle }_{\text{cg}}$ by their expressions assuming absence of correlations: [FG]≈4[F][G] and ${\langle [\text{FG}]\rangle }_{\text{cg}}\;\approx \;{\langle [\text{FG}]\rangle }_{\text{cg}}^{u}$ for given [F]. The function ${\langle [\text{FG}]\rangle }_{\text{cg}}^{u}\left(\left[F\right]\right)$ is the quantity given in Eq. **7** for uniformly randomly placed forest (see orange curve in *SI Appendix*, Fig. S2 *A*–*C*). Eq. **13** is bistable with stable high and low forest cover steady states over a wide range of parameters. It accurately predicts the location of both stable steady states (blue line in *SI Appendix*, Fig. S3). Yet, its prediction of the threshold (unstable) steady state and the dynamics remains strongly biased. Indeed, away from the stable steady states, the interplay of forest demography and fire-induced forest erosion creates landscapes in which forest is spatially aggregated, strongly violating the assumption of the absence of correlations. This can be seen in *SI Appendix*, Fig. S2 *A*–*C*. which shows that for given forest area [F], the forest perimeter of simulations, [FG], lies below that predicted by the mean-field, ${\left[\mathrm{FG}\right]}_{\mathrm{mf}}$, i.e.,

[14] $$\left[\mathrm{FG}\right]<{\left[\mathrm{FG}\right]}_{\mathrm{mf}}=4\left[F\right]\left(1-\left[F\right]\right),$$

implying that forest is more spatially aggregated than assumed in the mean field. Aggregation results from forest spreading close to existing forest and from lower survival of forest cells that are more exposed to fire (i.e., less aggregated). Forest gain is smaller when aggregated (*SI Appendix*, Fig. S2 *D*–*F*) due to the smaller perimeter. Aggregation reduces forest loss at low cover while it increases forest loss at high cover (*SI Appendix*, Fig. S2 *D*–*F*). This is so because aggregation makes forest cells individually less exposed to fire but collectively less effective at blocking fires, where the individual effect is dominant at low cover and the collective effect is dominant at cover values near and above the fire percolation threshold.

For our choice of parameters, the stable steady states and the lower saddle-node bifurcation contain negligible spatial structure, such that mean-field predictions are accurate. We derive their expressions below for β≈0.

### Stable Steady States.

By Eq. **13**, the low-cover steady state ${\left[F\right]}_{-}^{*}$ has to be approximately zero for β≈0. At high forest cover, loss due to fire is negligible, such that the high-cover steady state ${\left[F\right]}_{+}^{*}$ can also be obtained from Eq. **13** (using β≈0):

[15] $${\left[F\right]}_{-}^{*}=0,\;{\left[F\right]}_{+}^{*}=1-\frac{\gamma }{4\alpha }.$$

For the chosen parameters, we have ${\left[F\right]}_{+}^{*}\;=\;0.83$, which is in excellent agreement with simulations (Fig. 2).

### Onset of Bistability.

At low forest cover, grass consists of a single cluster, such that we can write in Eq. **13**${\langle [\text{FG}]\rangle }_{\text{cg}}^{u}\;=\;\left[\mathrm{FG}\right]\;=\;$4[F][G]. Hence (when β≈0):

[16] $$\frac{\text{d}}{\text{d}t}\left[\text{F}\right]=4\alpha \left[\text{F}\right]\left[\text{G}\right]-\gamma \left[\text{F}\right]-4\phi Np_{\text{f}}{\left[\text{G}\right]}^{2}\left[\text{F}\right].$$

An expression for the lower bifurcation point can be obtained by finding the root of the derivative of the right-hand side of Eq. **16** with respect to [F] at $\left[F\right]\;=\;{\left[F\right]}_{-}^{*}\;=\;0$, giving the relation $4\alpha -\gamma -4\phi Np_{f}\;=\;0$. Rearranging this relation for ϕN, we can obtain the fire ignition rate above which tropical forests are bistable with grasslands:

[17] $${\left(\phi N\right)}_{\min}=\frac{1}{p_{f}}\left(\alpha -\frac{\gamma }{4}\right).$$

For the chosen parameters, ${\left(\phi N\right)}_{\min}\;=\;0.25$, which agrees well with simulations (Fig. 2) and which corresponds to a maximum fire return interval of ${\left(\phi N\right)}_{\min}^{-1}\;=\;4\;y$.

## Discussion

In this paper, we showed how nonlinear dynamics and bistability of tropical forest emerge spontaneously from the patch-scale rules of forest and fire spread, without assuming equations or thresholds for the effects of fire as in previous work. Below, we first summarize our main results on structure and dynamics. Then, we discuss the importance of the emergent structure as indicated by comparison with mean-field approximations. Finally, we highlight the potential practical implications of our results for resilience assessment and conservation.

### Emergent Structure and Dynamics.

Our simulations showed that spatial structure emerges due to forest expansion and fire-induced damage at the forest perimeter. As a consequence, the forest perimeter appeared in both the gain and loss side of our landscape-scale balance equation of forest area, Eq. **9**, where losses require weighting by adjacent grassland area. Remarkably, when plotting the changes predicted by our balance equation versus forest area, using landscapes from the simulations, we found that they lie on an approximate curve (Fig. 4 *G* and *H*). As this curve shows the change of forest area as a function of forest area, this means that the emergent macroscopic dynamics can be described by a simple ODE, Eq. **10**. In this emergent closed form of our balance equation, the perimeter quantities determine the nonlinearities. Therefore, Eq. **10** elucidates how forest dynamics and bistability are linked to the forest geometry that emerges from the patch-scale spreading rules. Note that, as in previous work, Eq. **10** does not include fire explicitly because it does not contain equations for fire. This follows from timescale separation between fire and vegetation dynamics, an assumption that was already implicit in mean-field models (12, 17–19, 21); mean field in refs. 10 and 15] and microscopic models (microscopic models in refs. 10 and 15) focusing on alternative stable states. However, previous work derived the implicit effect of fire in closed form by relying on standard percolation theory, which assumes that occurrence of flammable patches is spatially uncorrelated (25). As we did not rely on percolation theory but observed the closed form emerging in simulations (Fig. 4), we could avoid the biases that affect previous work.

### Evaluation of Mean-field Models.

We compared mean-field models against the emergent closed form of our balance equation to assess their validity (*SI Appendix*, Fig. S2) and to show where spatial structure is important. This showed that mean-field models are in qualitative but not quantitative agreement with simulations: Existence of bistability, but not its parameter range, is robust to mean-field assumptions. In particular, the simple mean field (*SI Appendix*, Eq. **S7**) is strongly biased due to its failure to account for two phenomena that are present in the microscale model: i) spontaneous fire extinction on the fast timescale, leading to separated rapid fire spreading events, ii) nearest-neighbor spreading of fire and forest, leading to emergent aggregation of forest patches away from the steady states. The former violates the mean-field assumption of large system size (N→∞) and the latter that of absence of correlations. That spatial structure affects steady states and dynamics is well known (e.g., refs. 22 and 24). Even when addressing timescale separation and using results from percolation theory for the effect of fire (*SI Appendix*, Eqs. **S18** or **S22**), a large bias remains except near the alternative stable states *SI Appendix*, Fig. S2. This is because standard percolation theory only considers lattice configurations with uniform random (i.e., spatially uncorrelated) placement of flammable sites, while our forest-grass landscapes are shaped by past fires and vegetation dynamics. As Fig. 3 *A* and *B* (at t=0) shows, forest aggregation is particularly strong at the tipping threshold for forest collapse, implying that mean-field models cannot be used to study abrupt forest dieback. Despite their severe bias concerning forest dynamics and tipping, mean-field models are still useful for studying regimes with little structure, such as near the stable equilibria or for dynamics with uniform seed dispersal. This enabled us to derive expressions for these equilibria (Eq. **15**) and the point of onset of bistability (Eq. **17**). The latter result was not obtained by previous mean-field models because they did not include parameters that relate directly to fire (10, 12), see *SI Appendix*, section S7 for a suggested modification] or they did not account for timescale separation in finite domains (16).

### Implications for Resilience Assessment and Conservation.

The link between geometry and dynamics implies that tropical forest resilience can be empirically estimated from its spatial structure. The spatial structure, as captured by the perimeter quantities [FG] and ${\langle [\text{FG}]\rangle }_{\text{cg}}$, can hence be treated as a measurable parameter additional to the microscopic parameters. Microscopic parameters (given in Table 1) can be inferred from remote-sensed data (as in ref. 30) or from experiments (as for fire spread in ref. 11), while the perimeter quantities can be calculated for any observed landscape. In regimes with negligible spatial structure, one can assess stability or resilience from the microscopic parameters alone, based on mean-field results. For example, if the onset point of bistability at low tree cover lies in the regime without spatial structure, as in our simulations, one can directly estimate the minimum fire ignition rate for onset of bistability from the microscopic parameters (Eq. **17**). This expression then shows which natural or abandoned degraded areas of low tree cover with fire ignition rate beyond this point will not spontaneously recover to closed tropical forest. In regimes with spatial structure, the mean field is highly inaccurate (*SI Appendix*, Fig. S2), such that spatial structure needs to be considered in addition to the parameters. In particular, in our simulations, the tipping threshold obtains spatial structure at higher fire ignition rates (Fig. 3 *A* and *B* at t=0) and approaches the stable forest equilibrium much more closely than in the mean field (*SI Appendix*, Fig. S3). While this makes the mean field unsuitable for studying forest resilience and dieback, our balance equation (Eq. **9**) does not have this limitation because it makes no assumption on spatial structure. We demonstrated how Eq. **9** permits estimation of the resilience of a landscape to perturbations, via $\lambda_{F}$ (Eq. **12**). In contrast to generic indicators of resilience (31, 32), $\lambda_{F}$ is an indicator that can be obtained from a single landscape and for which the contribution of each relevant spatial process can be examined. Furthermore, landscape perturbations by human intervention can be evaluated, through sensitivity $\lambda_{F}$, for how they will amplify or mitigate fire-vegetation feedback. Forest conservation/restoration may introduce targeted perturbations that most efficiently prevent resilience loss of high-cover states or induce resilience loss of low-cover states. For instance, in Fig. 5*A*, forest dieback is averted by a perturbation that divides the largest grass cluster into smaller ones. It may thus be anticipated that maintenance or creation of barriers to fire spread will be essential here.

Future work could explore further realism, such as environmental heterogeneity, longer dispersal ranges, nonlattice geometry (as in ref. 15), inclusion of other tree types (such as in savanna dynamics: refs. 10, 12, 15, 17, and 33–38), or vegetation-rainfall feedback (39). This may result in additional relevant quantities in Eq. **9**. Additionally, larger domain sizes may lead to more gradual transitions on the macroscopic scale (40) and *SI Appendix*, section S6].

## Materials and Methods

### Details of the FGBA Probabilistic Cellular Automaton.

The FGBA probabilistic cellular automaton is a minimal spatial stochastic process that models the joint dynamics of tropical vegetation and fire. It is an adapted version of the BGT(A) model of ref. 16. The modifications compared to ref. 16 are the following: i) it runs in continuous time, ii) it includes a spontaneous forest mortality rate γ, iii) species T is labeled as F, consistent with other models of tropical vegetation dynamics (12, 15, 18). Note that according to some definitions, probabilistic continuous-time cellular automata are considered interacting particle systems. In general, when studying the stochastic dynamics of a number n of interacting species on a square lattice with N cells, the state of the system can be represented as

$$X:=(X_{1},X_{2},...,X_{N}),$$

where $X_{i}$ is the label of the species that occupies cell i. Each cell is occupied by exactly one of four possible species: grass, forest, burning, and ash, with labels G, F, B, and A, respectively. Transitions between states (species) occur in continuous time, resulting in a continuous-time Markov chain with a state space of size $n^{N}$. The reaction rules for transitions between states are shown in Table 1, where spontaneous conversions are shown on the left and conversions due to nearest-neighbor interactions on the right (see also Fig. 1*A*).

The latter type of interaction occurs over each four nearest neighbor connections of the indicated type. For example, fire will spread into a given grass cell with a rate $\rho_{g}$ for each burning neighbor. For realistic timescales, our parameters satisfy Eqs. **1** and **2**, which were empirically justified in ref. 16. We borrow our notation from the moment closure literature (e.g., refs. 22–24 and 41), writing the global fraction of species with label x and the interface between species with label x with label y, respectively, as

[18] $$\left[x\right]:=\frac{1}{N}\sum_{i}^{N}\delta_{x}\left(X_{i}\right),\;\left[\mathrm{xy}\right]:=\frac{1}{N}\sum_{i,j}^{N}A_{\mathrm{ij}}\delta_{x}\left(X_{i}\right)\delta_{y}\left(X_{j}\right),$$

where both are normalized by N, δ is the Kronecker delta function ($\delta_{x}\left(y\right)\;=\;1$ if y=x and 0 otherwise) and $A\in {\left\{0,1\right\}}^{N\times N}$ the adjacency matrix. We simulated the cellular automaton via a Gillespie algorithm (42) and used a domain of N=100×100 (N=200×200 in Fig. 1) cells with periodic boundary conditions.

### Noninvasive Feedback Control.

To study steady states regardless of their stability in a simulation, we apply noninvasive feedback control (43–46). To obtain the dependence of equilibria of [F] on fire ignition rate ϕ, we introduced an artificial stabilizing feedback loop of the form

[19] $$\phi \left(t\right)=\phi_{0}+g\left(\left[F\right]\left(t\right)-{\left[F\right]}_{\mathrm{ref}}\right).$$

**Fig. 6.**
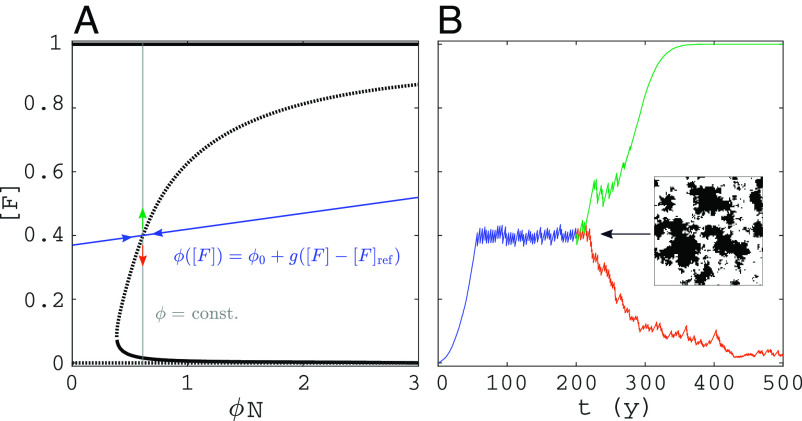
Feedback control applied to the cellular automaton without spontaneous mortality (γ=0): (*A*) The unstable steady state of the bifurcation diagram (dashed) was derived via feedback control by letting ϕ be a function of [F] (blue line) such that it is stabilized, then obtaining (ϕ,[F]) by averaging and repeating this for many ${\left[F\right]}_{\mathrm{ref}}$ (with appropriate g), (*B*) a regular simulation with the same ϕ value [solid gray in (*A*)] and starting from the final state of the controlled simulation tips up or down depending on initial perturbations, (*B*) snapshot of the domain at the saddle for the control indicated in (*A*) (black: forest, white: grass). For other parameters, see Table 1.

The factor g is called the feedback control gain and is problem specific. The property of noninvasiveness means that the controlled simulations have the same equilibria as regular simulations (47–49). This implies that if one extracts the equilibrium values of the controlled simulation $\left(\phi^{*},{\left[F\right]}^{*}\right)$, one can use them to plot a 1-parameter bifurcation diagram of the simulation without control. Fig. 6 shows the control graphically. The feedback Eq. **19**, indicated in blue in Fig. 6*A*, stabilizes a steady state that is unstable in a regular simulation. This can be seen in Fig. 6*B*, where the unstable steady state is first stabilized via control, after which the control is removed and a regular simulation is started with the effective rate and the landscape (*Inset*) obtained from the controlled simulation. Depending on initial perturbations, the regular simulation gets either attracted to the 100% forest state or to the low tree cover state. When the controlled simulation is in equilibrium (steady part of the blue curve in Fig. 6*B*), the steady state values of [F] are obtained via taking the time average, i.e.,

[20] $$\bar{\left[F\right]}=\frac{1}{T}\int_{t_{0}}^{t_{0}+T}\left[F\right]\left(t\right)dt,$$

where $t_{0}$ is the time after which the dynamics have settled to a steady state and T the averaging time. If $n_{G\to B}$ is the number of ignition events between $t=t_{0}$ and $t=t_{0}+T$, the steady state of ϕ is obtained by calculating the mean ignition rate as $n_{G\to B}/T$ and dividing this by the mean number of grass cells, such that

[21] $$\overset{¯}{\phi }=\frac{n_{G\to B}/T}{\bar{\left[G\right]}},$$

where $\bar{\left[G\right]}$ is obtained as in Eq. **20**. When repeating this exercise for many ${\left[F\right]}_{\mathrm{ref}}$ values, one can get multiple points on the unstable branch. Points on the stable branches can be obtained with regular simulations. On the final selection of points, we applied Gaussian process regression to obtain smooth curves and used moving block bootstrapping (50) to obtain confidence intervals. One of the advantages of applying control is that one can obtain states for which one would have to wait prohibitively long in a regular simulation due to their instability.

### Forest Loss due to a Single Fire.

A fire in grassland cluster with index j that reaches its interface with adjacent forest induces a forest loss that can be approximated as follows. Consider a single forest cell i located at the interface with grassland cluster j with ${\left[\mathrm{FG}\right]}_{i,j}$ number of neighboring grass cells. When assuming that spreading events are independent, the probability that the forest cell gets burnt is the complement of the probability that none of its neighboring grass cells in cluster j spread the fire to the forest cell:

[22] $$q_{i,j}:=1-{\left(1-p_{f}\right)}^{{\left[\mathrm{FG}\right]}_{i,j}}\approx p_{f}{\left[\mathrm{FG}\right]}_{i,j},$$

where the approximation on the right is valid for small $p_{f}$. Summing over all forest cells at the interface of grassland cluster j, we obtain the expected loss of forest per fire event as shown in Eq. **4**:

[23] $$\Delta_{F,j}^{\mathrm{loss}}:=\sum_{i}q_{i,j}=p_{f}{\left[\mathrm{FG}\right]}_{j}.$$

This approximation also assumes that burning forest cells at the interface do not spread the fire further, which also relies on $p_{f}$ being small. For an evaluation of the validity of this approximation in case of landscapes without spatial structure, see *SI Appendix*, Fig. S7.

### Critical Hole Size for an Abrupt Shift When γ=β=0.

When there are no spontaneous transitions and we perturb a fully closed forest of 100% cover by creating a hole with grassland, an expression can be obtained for the critical hole size beyond which fire causes an abrupt shift to grassland. Using that grassland is a single cluster, Eq. **9** becomes

[24] $$\frac{d[F]}{dt}=\left(\alpha -\phi Np_{f}\left[G\right]\right)\left[\mathrm{FG}\right],$$

which has two absorbing steady states ${\left[F\right]}_{l}^{*}\;=\;0$ and ${\left[F\right]}_{h}^{*}\;=\;1$, and an unstable steady state at ${\left[F\right]}_{c}^{*}\;=\;1-\frac{\alpha }{\phi Np_{f}}$. The critical hole size is then the complement of the unstable steady state:

[25] $${\left[G\right]}_{c}=\frac{\alpha }{\phi Np_{f}},$$

which can also be written as ${\left[G\right]}_{c}=\phi_{1}/\phi$, where $\phi_{1}$ is the value of ϕ for which ${\left[G\right]}_{c}=1$, or also the lower limit of the bistability range.

### Sensitivity to Perturbations.

We estimated ${\overset{¯}{\lambda }}_{F}$ in Fig. 5d for a given landscape by averaging Eq. **12** over realizations of different types of perturbations:

[26] $${\overset{¯}{\lambda }}_{F}=\langle \frac{\sum_{i}w_{i}\Delta \dot{\left[F\right]}\left(X,{\left(\delta X\right)}_{i}\right)}{\sum_{i}w_{i}\Delta \left[F\right]\left(X,{\left(\delta X\right)}_{i}\right)}\rangle .$$

Each of ${\left(\delta X\right)}_{i}$ are perturbations that one might expect in simulations, such as removal of a fraction of perimeter forest cells in the largest grass cluster or spontaneous mortality/growth of forest. The weights $w_{i}$ are determined by the rates/probabilities of occurrence of the perturbations. The ⟨·⟩ denote an average over 64 realizations.

## Supplementary Material

Appendix 01 (PDF)

## Data Availability

Algorithm data have been deposited in Github (https://github.com/b-wuyts/fgba) (51).
