# Supplementary material for: Emergent structure and dynamics of tropical forest-grassland landscapes
Source: Proc Natl Acad Sci U S A. 2023 Oct 30;120(45):e2211853120. doi: 10.1073/pnas.2211853120 (PMC10636392; doi:10.1073/pnas.2211853120)
Supplement: Supplementary file 1 — Appendix 01 (PDF) [file pnas.2211853120.sapp.pdf]

Supporting Information for

Emergent structure and dynamics of tropical forest-grassland landscapes

Bert Wuyts, Jan Sieber

Centre for Systems, Dynamics and Control, University of Exeter, EX4 4QF, UK

[1]To whom correspondence should be addressed. E-mail: b.wuyts@ex.ac.uk

This PDF file includes:

Figs. S1 to S12
SI References

Contents

S1 Supplementary figures referenced from the main text 2

S2 Relevant characteristics of the fire spreading process 3
- A Definition and mean field . . . 3
- B Extinction in finite systems . . . 4
- C Percolation analysis of a single fire event . . . 4

S3 Simple mean field of joint forest and fire spread 5

S4 Two-timescale mean field of joint forest and fire spread 6
- A Well-mixed fire and forest . . . 6
  - A.1 Fast process: forest loss due to a single fire . . . 6
  - A.2 Slow processes: forest demography and fire damage . . . 7
- B Spatial fire percolation and uniformly randomly placed forest . . . 7
- C Detailed comparison against simulations . . . 9

S5 Evolution of fronts — heterogeneous states 9

S6 A note on finite sizes and bistability 11

S7 How to include fire parameters in existing mean-field models 11

S8 Additional figures 13

S1. Supplementary figures referenced from the main text

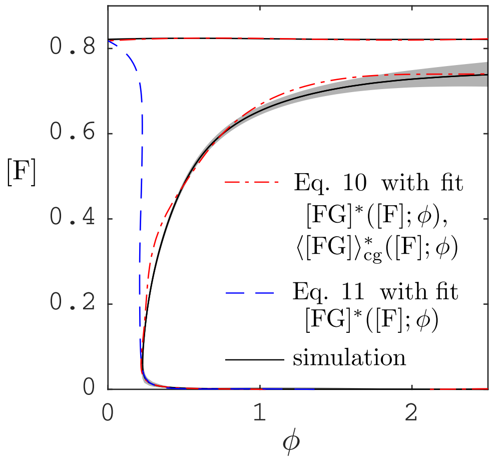

Fig. S1. Steady states of Eq. 10 (multicluster – dot-dashed red) and of Eq. 11 (single cluster – dashed blue) compared to controlled simulations (solid black with shading indicating two-standard deviation confidence interval of the mean). Parameters in the cellular automaton: $\gamma{=}0.02$, $\alpha{=}0.03$, $\beta{=}2\cdot10^{-4}$, $\rho_{\mathrm{g}}{=}9\cdot10^{6}$, $\rho_{\mathrm{f}}{=}1.11\cdot10^{5}$, $\mu{=}10^{6}$, $\lambda{=}5$.

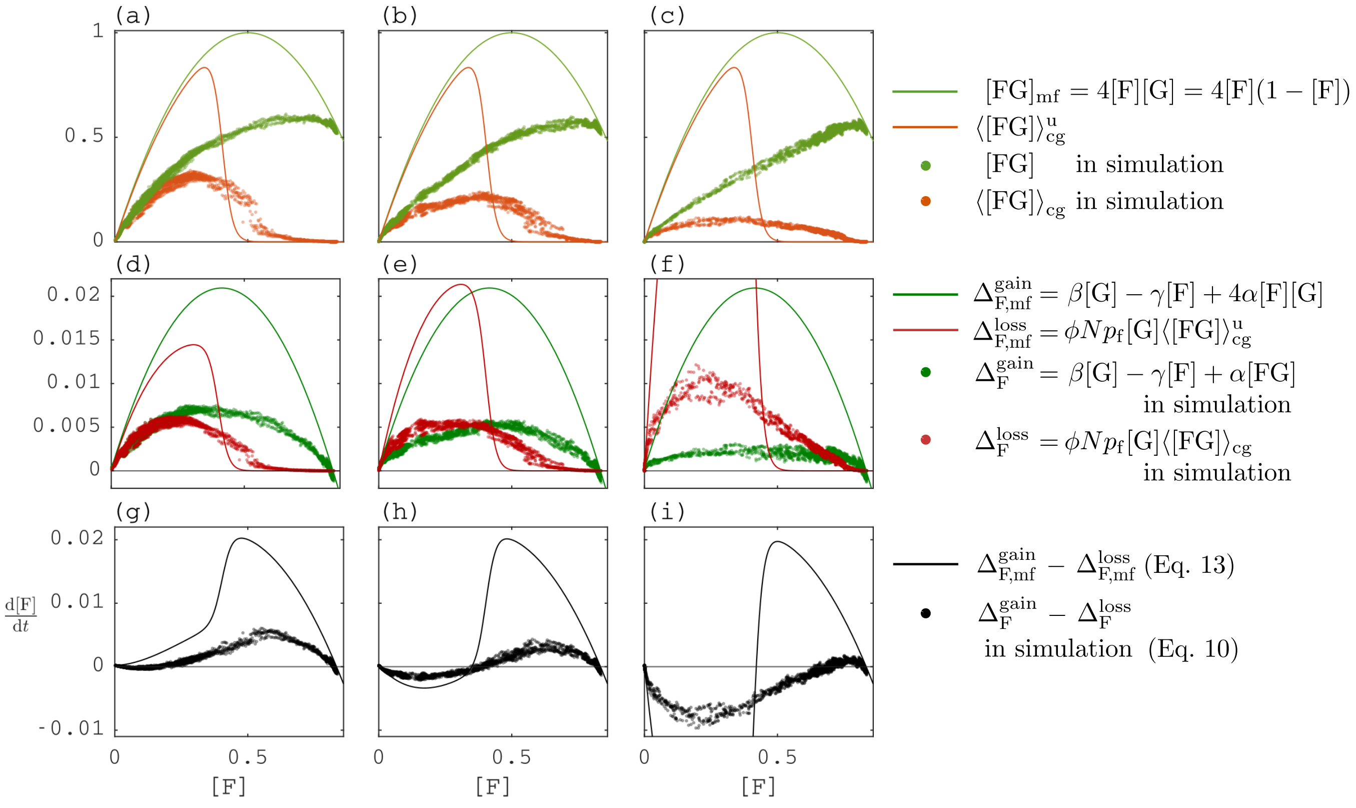

Fig. S2. Emergent relations between key quantities and forest area [F] compared to the mean field with site percolation (dots: simulations, lines: corresponding mean field quantities from Section S4B): (a–c) forest perimeter [FG] (green) and grassland-weighted forest perimeter $\langle[\mathrm{FG}]\rangle_{\mathrm{cg}}$ (orange), where green line equals 4[F][G] and orange line was called $\langle[\mathrm{FG}]\rangle^{u}_{\mathrm{cg}}$ in the main article, (d–f) forest gain terms and loss terms in Eqs. 5 and 6, (g–i) forest area rate of change $(\mathrm{d}/\mathrm{d}t)[\mathrm{F}]$ from Eq. 9. Columns correspond to vertical dashed lines in Fig. 2 ($\phi N{=}0.257$, $\phi N{=}0.38$, $\phi N{=}1.32$). Simulation results are identical to Fig. 4. Domain size: $N{=}100$x100 cells. See Section S4B for details of derivation for $\langle[\mathrm{FG}]\rangle^{\mathrm{u}}_{\mathrm{cg}}$ and $[\mathrm{FG}]_{\mathrm{mf}}$.

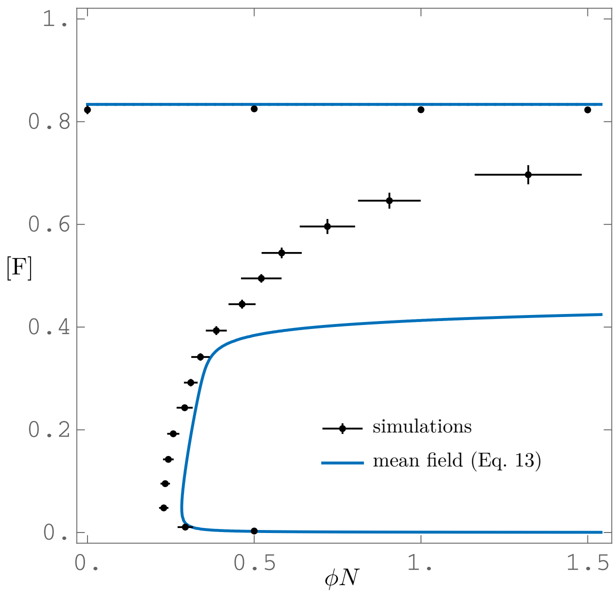

Fig. S3. Comparison of steady state forest as a function of ignition rate in the time-separated mean-field and in simulations (dots with error bars: simulations, lines: mean field). A large difference between mean-field model and simulation occurs for the threshold steady state, while the mean-field model is accurate for high- and low-tree-cover alternative stable states. See Section S4B and Fig. S9 for comparison of other scenarios and mean-field approximations.

S2. Relevant characteristics of the fire spreading process

Before obtaining the mean-field equations for coupled vegetation and fire dynamics, we analyse the fire spreading process in isolation. The insights from this section will enable us to set up a mean-field model that constitutes the fairest comparison against the analysis in the main text.

A. Definition and mean field. When we remove state F and its conversion rates to/from other types $(\alpha, \beta, \gamma, \rho_{\rm f})$ from the FGBA process, the dynamics show fire spread alone. We call this the *GBA process*. Writing x_i as shorthand for $\delta_{\mathrm{x}}(X_i)$ (equalling 1 if $X_i = \mathrm{x}$ and 0 otherwise) and taking expectations in each cell i, we obtain equations for the rate of change of the expectation that cell i is occupied by species $x \in \{\mathrm{G}, \mathrm{B}, \mathrm{A}\}$,

$$\begin{aligned} \frac{\mathrm{d}}{\mathrm{d}t}\big\langle \mathrm{G}_i \big\rangle &= \lambda \big\langle \mathrm{A}_i \big\rangle - \Big\langle \mathrm{G}_i\big(\phi + \sum_{j\in\mathcal{N}(i)} \rho_{\mathrm{g}} \mathrm{B}_j\big)\Big\rangle, \\ \frac{\mathrm{d}}{\mathrm{d}t}\big\langle \mathrm{B}_i \big\rangle &= \phi \big\langle \mathrm{G}_i \big\rangle - \mu \big\langle \mathrm{B}_i \big\rangle + \Big\langle \rho_{\mathrm{g}} \mathrm{G}_i \sum_{j\in\mathcal{N}(i)} \mathrm{B}_j \Big\rangle, \\ \frac{\mathrm{d}}{\mathrm{d}t}\big\langle \mathrm{A}_i \big\rangle &= -\frac{\mathrm{d}}{\mathrm{d}t}\big\langle \mathrm{G}_i \big\rangle - \frac{\mathrm{d}}{\mathrm{d}t}\big\langle \mathrm{B}_i \big\rangle = \mu \big\langle \mathrm{B}_i \big\rangle - \lambda \big\langle \mathrm{A}_i \big\rangle, \end{aligned} \quad \underset{\frac{1}{N}\sum_i^N}{\Longrightarrow} \quad \begin{aligned} \frac{\mathrm{d}}{\mathrm{d}t}\langle[\mathrm{G}]\rangle &= \lambda\langle[\mathrm{A}]\rangle - \phi\langle[\mathrm{G}]\rangle - \rho_{\mathrm{g}}\langle[\mathrm{GB}]\rangle, \\ \frac{\mathrm{d}}{\mathrm{d}t}\langle[\mathrm{B}]\rangle &= \phi\langle[\mathrm{G}]\rangle - \mu\langle[\mathrm{B}]\rangle + \rho_{\mathrm{g}}\langle[\mathrm{GB}]\rangle, \\ \frac{\mathrm{d}}{\mathrm{d}t}\langle[\mathrm{A}]\rangle &= \mu\langle[\mathrm{B}]\rangle - \lambda\langle[\mathrm{A}]\rangle, \end{aligned} \qquad \text{[S1]}$$

where $\langle\cdot\rangle$ are ensemble averages, [x] the domain fraction of species x, and [xy] the total number of neighbouring xy pairs divided by N, later referred to as the xy interface or xy perimeter. This set of equations can be derived rigorously from the master equation (e.g. 1). To go from individual (left) to population level (right), we summed over i and divided by N, using Eq. 18. Equation S1 is not a closed system. To close the system, we need to determine all undetermined terms [xy] on the right-hand side without creating new unknowns. The simplest way to do this is to assume absence of pairwise correlations, i.e. $\langle[\mathrm{xy}]\rangle = 4\langle[\mathrm{x}]\rangle\langle[\mathrm{y}]\rangle$. We take the additional assumption of $N \to \infty$, such that the law of large numbers applies and $[\mathrm{x}] \to \langle[\mathrm{x}]\rangle$. These assumptions are valid when all cells in an large domain interact with each other at uniform contact rates of order $1/N$. This results in the *simple mean-field approximation* of the GBA process:

$$\begin{aligned} [\dot{\mathrm{G}}] &= \lambda[\mathrm{A}] - \phi[\mathrm{G}] - 4\rho_{\mathrm{g}}[\mathrm{G}][\mathrm{B}], \\ [\dot{\mathrm{B}}] &= \phi[\mathrm{G}] - \mu[\mathrm{B}] + 4\rho_{\mathrm{g}}[\mathrm{G}][\mathrm{B}], \\ [\dot{\mathrm{A}}] &= \mu[\mathrm{B}] - \lambda[\mathrm{A}], \end{aligned} \qquad \text{[S2]}$$

where we also used the dot notation for time derivatives. Substituting $[\mathrm{A}] = 1 - [\mathrm{G}] - [\mathrm{B}]$ and taking only the independent equations, we finally obtain

$$\begin{aligned} [\dot{\mathrm{G}}] &= \lambda(1 - [\mathrm{G}] - [\mathrm{B}]) - \phi[\mathrm{G}] - 4\rho_{\mathrm{g}}[\mathrm{G}][\mathrm{B}], \\ [\dot{\mathrm{B}}] &= \phi[\mathrm{G}] - \mu[\mathrm{B}] + 4\rho_{\mathrm{g}}[\mathrm{G}][\mathrm{B}]. \end{aligned} \qquad \text{[S3]}$$

We further focus on the case $\phi = 0$, the reason for which will become clear below. When $\phi = 0$, Eq. S3 has two steady states, a trivial one at $([\mathrm{G}], [\mathrm{B}]) = (1, 0)$ and one at $([\mathrm{G}], [\mathrm{B}]) = (\frac{\mu}{4\rho_{\mathrm{g}}}, \frac{1-\mu/4\rho_g}{1+\mu/\lambda})$. The eigenvalues of the Jacobian of Eq. S3 show that for $4\rho_{\mathrm{g}}/\mu > 1$, the trivial steady states is a saddle and the non-trivial a spiral sink. For $4\rho_{\mathrm{g}}/\mu < 1$, the trivial state state is a stable

node and the only physical solution. Hence, the steady states exchange stability at the transcritical bifurcation at $4\rho_g/\mu = 1$. The GBA process with $\phi = 0$ is equivalent to the SIRS spreading process in epidemiology (2), which represents spread of a disease in a population with waning immunity. Fire B plays the role of infected individuals. Infections spread through a population of susceptibles G at rate ρ_g per GB link. They subsequently acquire a state of immunity A at rate μ, which can be lost at rate λ. The non-trivial steady state corresponds to the endemic equilibrium and the transcritical bifurcation to the epidemic threshold R_0.

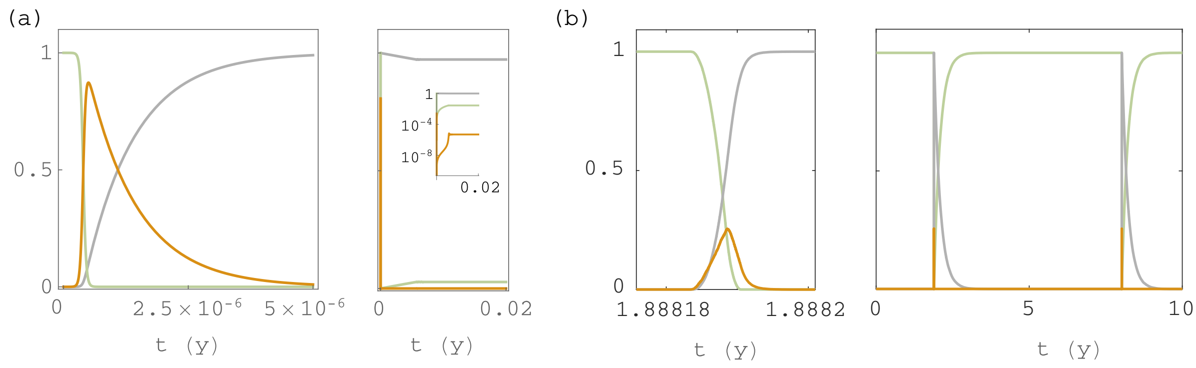

Fig. S4. Time series of the GBA process ($\lambda, \phi \neq 0$): (a) mean-field approximation, (b) simulation on a square lattice. G: green, B: orange, A: grey.

B. Extinction in finite systems. A well-known characteristic of this spreading process with $\phi = 0$ and $[\mathrm{B}]_0 > 0$ is that in finite systems, it goes extinct in finite time, even when $R_0 > 1$ (3). This is so because stochastic excursions away from the non-trivial equilibrium will eventually reach the absorbing trivial state. When the spontaneous ignition rate $\phi > 0$ and the time to extinction is much smaller than the typical waiting time between ignition events, there are repeated fire events separated by extinction events. The dynamics then effectively behave as a series of GBA processes with $\phi = 0$ and $[\mathrm{B}]_0 > 0$. This is what we observe in cellular automaton simulations on a square lattice of 100×100 cells for realistic parameters (Fig. S4b, right panel). The mean-field approximation (Eq. S3), on the other hand, does not show extinction due to its assumption of $N \to \infty$. Instead, it shows a single pulse (Fig. S4a, left panel) after which a high-ash low-grass and non-zero fire steady state (the endemic equilibrium) is reached (Fig. S4a, right panel). In the case $\phi = 0$, the required lattice size to avoid extinction with high probability depends on the initial conditions (3), but for realistic parameter ranges, it is unrealistically large. This can be understood as follows.

- When the initial condition is a single fire, at least one cell has to keep on burning until the density of grass has regrown to a level sufficient for a new wave to propagate. This translates into the condition $(L/\Delta x)^2 \exp(-\mu/\lambda) \geq \mathcal{O}(1)$, such that $L \geq \mathcal{O}(10^{4 \cdot 10^4})$ for our parameters (taking a grid size of $\Delta x = 0.03$km as in (4)).
- When initial conditions are such that a band of the domain is immune at the start, a single fire can keep on burning by crossing the domain repeatedly (3). When assuming $\rho_g \gg \mu$ and using that waiting times between spreading events are exponentially distributed with mean $1/\rho_g$, a fire will spread throughout the domain in a time of the order $\tau \approx L/(\rho_g \Delta x)$. For there to be sufficient regrowth of grass on this time scale, we need $L/(\rho_g \Delta x) \approx 1/\lambda$, or $L \approx \rho_g \Delta x/\lambda$. For the parameters we have chosen, this means $L = \mathcal{O}(10^4)$km, i.e. the order of magnitude of the earth's circumference, which is drastically smaller than the above estimate yet still impractically large.

Taking more conservative estimates for fire spreading rates or taking account of a small positive fire ignition rate $\phi = \mathcal{O}(\lambda/N)$ for the initial condition with a single burning cell, this may be decreased by an order of magnitude, i.e. the size of a continent or country. Still, in reality, extinction will occur on smaller scales due to spatiotemporal heterogeneity of forcing parameters as a consequence of climatic seasonality or existence of natural or artificial boundaries (such as forests), leading to a lower effective system size. Hence, in any real system, repeated extinction and system-scale oscillations are to be expected.

C. Percolation analysis of a single fire event. Therefore, a single fire in realistically sized systems corresponds to the case $\phi = 0$, starting with a single burning cell. Using that the regrowth of grass occurs on a much slower time scale, we can further also set $\lambda = 0$ in our following analysis. The GBA process with $\phi, \lambda = 0$ is equivalent to susceptible-infected-recovered (SIR) epidemic spreading (2). The final size of the epidemic in SIR epidemic spreading on a lattice shows a continuous phase transition (CPT) at a critical spreading rate ρ_g and scaling laws near the critical point obey those of the ordinary percolation universality class (5). Figure S5 shows mean quantities for SIR epidemic spreading on a square lattice in a range of infection probabilities and initial number of immune individuals, which are spatially uniformly distributed. In particular, we show that SIR epidemic spreading is a type of *mixed site-bond percolation*, with bond occupation probability given by $p_b := p_g = \rho_g/(\rho_g + \mu)$ (which is fixed at 0.9 in the main text, as in ref. (4)) and site occupation probability given by $p_s := [\mathrm{G}]_0$, i.e. the initial fraction of cells that are grass in fire spreading, or the complement of the initial fraction of immune individuals in epidemic spreading (with the rest being susceptible). In Fig. S5, we record the *mean cumulative probability of being burnt* $\langle Q \rangle$ and the *susceptibility* χ of $\langle Q \rangle$, defined as

$$Q := \frac{[\mathrm{A}]^* - [\mathrm{A}]_0}{[\mathrm{G}]_0}, \quad \text{[S4]} \qquad \chi = \frac{\langle Q^2 \rangle}{\langle Q \rangle}, \quad \text{[S5]}$$

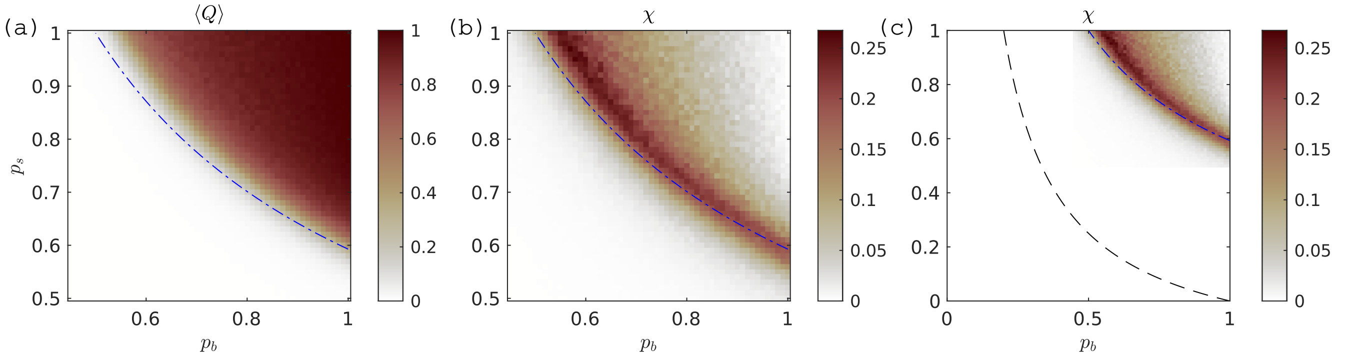

Fig. S5. GBA process with $\phi{=}\lambda{=}0$ (equivalent to square lattice SIR spreading): $\langle Q\rangle, \chi$ versus bond occupation probability $p_b := p_\mathrm{g}$ (Eq. 3) and site occupation probability $p_s{=}[\mathrm{G}]_0$. (a) Mean cumulative probability of being burnt $\langle Q\rangle$ (expectation of Eq. S4). (b) Susceptibility χ (Eq. S5). (c) Susceptibility χ compared to the mean-field percolation threshold $p_{s,\mathrm{mf}}$, given in Eq. S6 (dashed black). The dash-dotted blue line indicates the location of the infinite-size percolation threshold for uncorrelated mixed site-bond percolation (taken from (6)). The GBA model's percolation threshold lies at higher values (b) due to spatial correlation of p_g as explained in the text. The colour scale was taken from (7). See Fig. S11 for more detail.

where $[\cdot]_0$ denotes initial value and $[\cdot]^*$ final value. For $N{\to}\infty$, $\langle Q\rangle$ converges to the *percolation probability* P_∞, which is the probability that a grass cell belongs to the giant connected component. We use the location where χ peaks as an estimate of the percolation threshold (8, 9). When $p_s = [\mathrm{G}]_0 = 1$, we have pure bond percolation and when $\rho_\mathrm{g}/\mu \to \infty$, we have pure site percolation. The percolation threshold for standard mixed site-bond percolation (from (6)) is shown in Fig. S5 with a dot-dashed blue curve. The pure bond percolation threshold (when $[\mathrm{G}]_0 = 1$) of SIR epidemic spreading occurs at higher p_b than in standard bond percolation ($p_b \approx 0.538 > 0.5$) because the possibility of spreading to multiple neighbours makes the bond occupation probability spatially autocorrelated, as shown by (5). The pure site percolation limit shows the classical value (for the square lattice) of $[\mathrm{G}]_0 \approx 0.593$. From Eq. S3 (with $\phi = \lambda = 0$), we can obtain the mean-field percolation threshold $p_{s,\mathrm{mf}}$ by finding where the trivial state becomes unstable in Eq. S3, which is given by

$$4[\mathrm{G}]_0\frac{\rho_\mathrm{g}}{\mu} = 1 \implies p_{s,\mathrm{mf}} = [\mathrm{G}]_0 = \frac{1}{4}\left(\frac{1}{p_b} - 1\right). \quad \text{[S6]}$$

As shown in Fig. S5c, the mean-field approximation shows a large bias towards lower values.

Implications for the FGBA process On landscapes with forest, fires can be blocked (albeit imperfectly) by forest cells. These landscapes obtain a steady state shape due to the shaping processes of forest demography and fire. Hence, results from the spatially uniform $[\mathrm{G}]_0$ above do not apply to percolation effects in the full FGBA process. That is, when fire spreads on landscapes with forest, the critical point for pure site percolation ($\rho_\mathrm{g}/\mu \to \infty$, or $p_\mathrm{g} \to 1$) will in general depend not only on the site occupation probability $[\mathrm{G}]_0$ but also on the spatial correlation function of site occupation. For the idealised case of fire-proof forest ($p_\mathrm{f} = 0$), fire percolation on real landscapes is then equivalent to *correlated mixed percolation*, where correlations in bond occupation probability occur due to the spreading process, and correlations in site occupation probability occur due to the nonrandom spatial structure of the landscape. When $p_\mathrm{f} > 0$, the spreading process becomes a *heterogeneous (correlated) bond percolation processes*, i.e. a percolation process in which fire spread on grass occurs with bond occupation probability p_g and on forest with bond occupation probability p_f. The possibility of spreading on forest decreases the percolation thresholds compared to the correlated mixed percolation limit of $p_\mathrm{f} \to 0$. This decrease is expected to be small because forests do not spread fires well ($p_\mathrm{f} \approx 0$).

S3. Simple mean field of joint forest and fire spread

When we follow the same steps as in S2A, we obtain the *simple mean-field approximation* of the FGBA process:

$$\begin{aligned}
[\dot{\mathrm{G}}] &= \lambda[\mathrm{A}] - \phi[\mathrm{G}] - 4\rho_\mathrm{g}[\mathrm{G}][\mathrm{B}] - \beta[\mathrm{G}] - 4\alpha[\mathrm{F}][\mathrm{G}] + \gamma[\mathrm{F}],\\
[\dot{\mathrm{F}}] &= \beta[\mathrm{A}] + 4\alpha[\mathrm{F}][\mathrm{A}] - 4\rho_\mathrm{f}[\mathrm{F}][\mathrm{B}] + \beta[\mathrm{G}] + 4\alpha[\mathrm{F}][\mathrm{G}] - \gamma[\mathrm{F}],\\
[\dot{\mathrm{B}}] &= \phi[\mathrm{G}] - \mu[\mathrm{B}] + 4\rho_\mathrm{f}[\mathrm{F}][\mathrm{B}] + 4\rho_\mathrm{g}[\mathrm{G}][\mathrm{B}],\\
[\dot{\mathrm{A}}] &= \mu[\mathrm{B}] - \lambda[\mathrm{A}] - \beta[\mathrm{A}] - 4\alpha[\mathrm{F}][\mathrm{A}].
\end{aligned} \quad \text{[S7]}$$

The simple mean field shows bistability of tree cover (first shown by ref. (4) for $\gamma = 0$) in ranges of all parameters that are expected to show considerable spatial heterogeneity in a given ecosystem: $\alpha, \beta, \gamma, \phi$ (Fig. S6). However, despite being qualitatively correct, it shows a large bias compared to simulations. For the parameter ranges of our simulations, it has no

non-trivial solution for positive fire ignition rate ϕ, so we had to choose different parameter values to find its bistability range. This bias is due to the inability of the simple mean to capture two effects: repeated fire extinction on a fast timescale and the spatial nature of the two spreading processes. In the following section, we derive alternative mean-field models that partially correct for these biases.

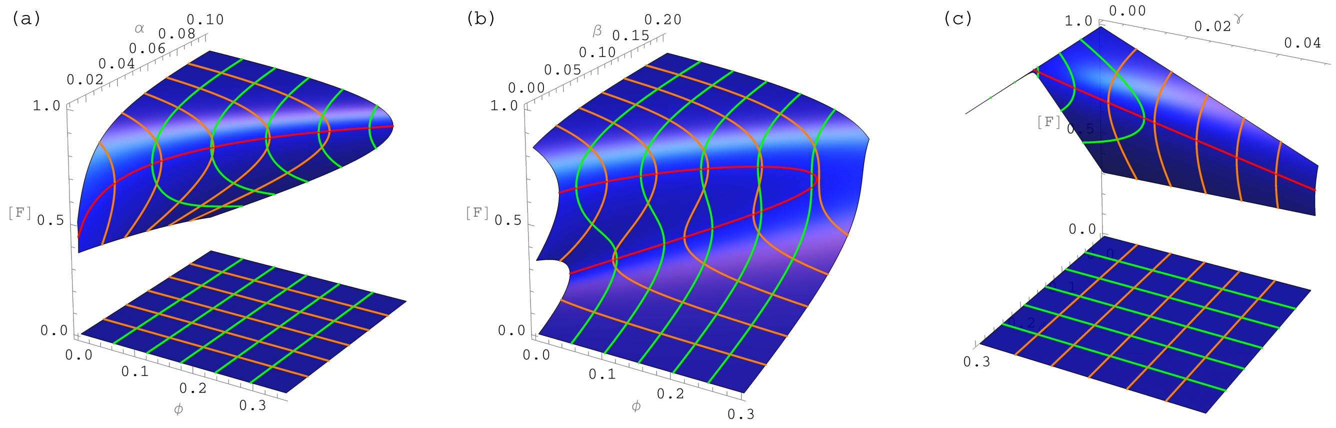

Fig. S6. Steady states and bifurcations of forest cover [F] in the simple mean field of the FGBA process (blue: steady state manifold, green/orange contours at fixed axis values, red: saddle-node bifurcations): (a) versus fire ignition rate ϕ and forest spreading rate α, (b) versus fire ignition rate ϕ and spontaneous forest growth rate β, (c) versus fire ignition rate ϕ and spontaneous forest mortality rate γ. Due to its large bias, the simple mean field shows different bistability ranges than the simulations. We set $p_{\rm g}=0.25$ (requiring a $\rho_{\rm g}$ that is 27x smaller than in simulations) such that bistability ranges are visible (remaining parameters are as in Table 1).

S4. Two-timescale mean field of joint forest and fire spread

Here, we derive an alternative mean-field model that takes account of separation of fire events in systems with realistic sizes, assuming that fire spread occurs on a much faster time scale than forest spread. This means that we can consider the fire spreading process in isolation with $\phi = \lambda = 0$ (as argued in Section S2), and take the asymptotic amount of forest burnt by a single fire before extinction on the fast time scale as forest mortality per fire event on the slow time scale.

A. Well-mixed fire and forest. We start with the simplest case, where both vegetation and fire mix uniformly, which is one way to conform with the mean-field assumption of absence of correlations.

A.1. Fast process: forest loss due to a single fire. On the fast time scale, we can set all small parameters related to forest demography, grass regrowth and fire ignition to zero (α=β=γ=λ=ϕ=0), such that we obtain

$$\begin{aligned}
\frac{\mathrm{d}}{\mathrm{d}t}[\mathrm{G}] &= -4\rho_{\rm g}[\mathrm{G}][\mathrm{B}],\\
\frac{\mathrm{d}}{\mathrm{d}t}[\mathrm{F}] &= -4\rho_{\rm f}[\mathrm{F}][\mathrm{B}],\\
\frac{\mathrm{d}}{\mathrm{d}t}[\mathrm{B}] &= -\mu[\mathrm{B}] + 4\rho_{\rm f}[\mathrm{F}][\mathrm{B}] + 4\rho_{\rm g}[\mathrm{G}][\mathrm{B}],\\
\frac{\mathrm{d}}{\mathrm{d}t}[\mathrm{A}] &= \mu[\mathrm{B}],
\end{aligned} \tag{S8}$$

where the products arise from the well-mixedness assumption as before. By rewriting the equations for $\frac{\mathrm{d}}{\mathrm{d}t}[\mathrm{G}]$, $\frac{\mathrm{d}}{\mathrm{d}t}[\mathrm{F}]$, $\frac{\mathrm{d}}{\mathrm{d}t}[\mathrm{A}]$ as

$$-\frac{1}{4\rho_{\rm g}[\mathrm{G}]}\frac{\mathrm{d}}{\mathrm{d}t}[\mathrm{G}] = -\frac{1}{4\rho_{\rm f}[\mathrm{F}]}\frac{\mathrm{d}}{\mathrm{d}t}[\mathrm{F}] = \frac{1}{\mu}\frac{\mathrm{d}}{\mathrm{d}t}[\mathrm{A}] = [\mathrm{B}], \tag{S9}$$

we can obtain [G] and [F] as a function of [A] via separation of variables and integration:

$$[\mathrm{G}](t) = [\mathrm{G}]_0 \exp\Big(-\frac{4\rho_{\rm g}}{\mu}[\mathrm{A}](t)\Big), \quad [\mathrm{F}](t) = [\mathrm{F}]_0 \exp\Big(-\frac{4\rho_{\rm f}}{\mu}[\mathrm{A}](t)\Big). \tag{S10}$$

Substituting Eq. S10 into the equation for $\frac{\mathrm{d}}{\mathrm{d}t}[\mathrm{A}]$ in Eq. S8 and setting the time derivative to zero, we obtain an implicit relation of the asymptotic amount of vegetation burnt:

$$[\mathrm{A}]^* = 1 - [\mathrm{G}]^* - [\mathrm{F}]^* = 1 - [\mathrm{G}]_0 \exp\Big(-\frac{4\rho_{\rm g}}{\mu}[\mathrm{A}]^*\Big) - [\mathrm{F}]_0 \exp\Big(-\frac{4\rho_{\rm f}}{\mu}[\mathrm{A}]^*\Big). \tag{S11}$$

When taking an initial state consisting of only grass and forest, that is $[\mathrm{G}]_0 = 1 - [\mathrm{F}]_0$, $[\mathrm{A}]^*$ can be found numerically as a function of ρ_g/μ, ρ_f/μ and $[\mathrm{F}]_0$. This can in turn be used to obtain the total amount of forest lost due to a single fire, from Eq. S10,

$$\Delta[\mathrm{F}]_\mathrm{mf} := [\mathrm{F}]_0 - [\mathrm{F}]^* = [\mathrm{F}]_0\Big(1 - \exp\big(-\frac{4\rho_\mathrm{g}}{\mu}[\mathrm{A}]^*\big)\Big), \qquad \text{[S12]}$$

where subscript mf denotes mean field. A plot of Equation S12 versus [F] is given in Fig. S7 (solid purple curve), which shows that the percolation threshold – where forest starts blocking fire – lies at unrealistically low grass cover, as was also the case for perfectly blocking forest (Fig. S5c, dashed line).

A.2. Slow processes: forest demography and fire damage. Now, we can define the mean-field forest gain and loss terms on the slow time scale as

$$\Delta^\mathrm{gain}_\mathrm{F,mf} := \beta[\mathrm{G}] + 4\alpha[\mathrm{F}][\mathrm{G}] - \gamma[\mathrm{F}], \qquad \text{[S13]}$$

$$\Delta^\mathrm{loss}_\mathrm{F,mf} := \phi N[\mathrm{G}]\Delta[\mathrm{F}]_\mathrm{mf}, \qquad \text{[S14]}$$

such that the final mean-field model becomes

$$\begin{aligned}\frac{\mathrm{d}}{\mathrm{d}t}[\mathrm{F}] &= \Delta^\mathrm{gain}_\mathrm{F,mf} - \Delta^\mathrm{loss}_\mathrm{F,mf}\\ &= \beta[\mathrm{G}] + 4\alpha[\mathrm{F}][\mathrm{G}] - \gamma[\mathrm{F}] - \phi N[\mathrm{G}]\Delta[\mathrm{F}]_\mathrm{mf},\\ &= \beta(1-[\mathrm{F}]) + 4\alpha[\mathrm{F}](1-[\mathrm{F}]) - \gamma[\mathrm{F}] - \phi N(1-[\mathrm{F}])[\mathrm{F}]\Big(1 - \exp\big(-\frac{4\rho_\mathrm{g}}{\mu}[\mathrm{A}]^*([\mathrm{F}])\big)\Big).\end{aligned} \qquad \text{[S15]}$$

The steady states are shown in Fig. S9 in solid purple for the same parameters as those used in the simulations of the main text. For low and high tree cover, it reproduces the steady states fairly accurately, but unlike the simulations, it shows no wide saddle in between. Hence, while this is an improvement compared to the simple mean field, there is still a large bias at intermediate tree cover. To address this bias, we need drop the assumption of uniform mixing for fire spread.

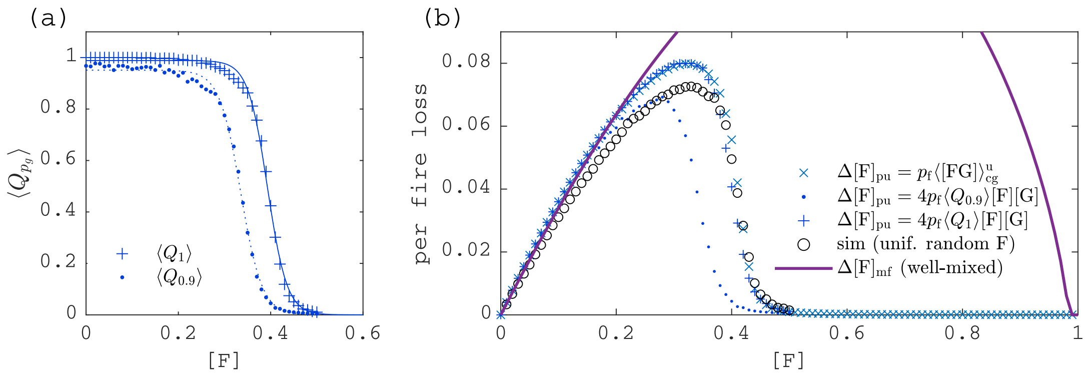

Fig. S7. Grass burning probabilities and forest loss per fire in landscapes without spatial structure: (a) probability that a grass cell burns $\langle Q_{p_\mathrm{g}}\rangle$, for $p_\mathrm{g} = 1$ ('+') and for $p_\mathrm{g} = 0.9$ ('·'), together with fits to logistic functions; (b) loss per fire estimated from grassland-weighted forest ('×', Eq. S16), from $\langle Q_{0.9}\rangle$ ('×', Eq. S20), from $\langle Q_1\rangle$ ('+', Eq. S20), by assuming uniform mixing (purple curve, Eq. S12), and measured in fire simulations with uniform random placement of forest ('∘').

B. Spatial fire percolation and uniformly randomly placed forest. While the uniform mixing assumption may be ecologically justified for forest spread in case of species with long-range seed dispersal, it is much harder to justify for fire spread, which is fundamentally a local contagion process. Therefore, we aim to take into account the effects of fire as a percolation process while still assuming absence of spatial correlations between forest cells. Because the percolation process affects forest loss, this only affects the loss function. Earlier mean-field models (10–12) accounted for the effects of fire percolation by making fire-affected rates threshold functions of tree cover, such as those shown in Fig. S7a, while assuming that vegetation remains spatially uncorrelated (for derivation, see 11). Therefore the mean-field analyses presented here provide the fairest points of comparison.

To estimate the loss due to fire, we will show two alternative approaches. The first is equivalent to our approach in the main text, using grassland-weighted forest perimeter to estimate exposed forest. The second estimates exposed forest via standard results from percolation theory, which are valid here due to the assumption of uniform random placement of forest.

1. Using the *grassland-weighted forest perimeter* $\langle[\mathrm{FG}]\rangle_\mathrm{cg}$ (see Eq. 7). According to this approach, fires spread perfectly to the forest perimeter, where a fraction of the forest is burnt. The difference with the main text is that the landscapes in which fire spreads have uniform random placement of forest. We indicate this difference below by the superscript u in $\langle[\mathrm{FG}]\rangle^\mathrm{u}_\mathrm{cg}$. The resulting loss per fire is

$$\Delta[\mathrm{F}]_\mathrm{pu} = p_\mathrm{f}\langle[\mathrm{FG}]\rangle^\mathrm{u}_\mathrm{cg}, \qquad \text{[S16]}$$

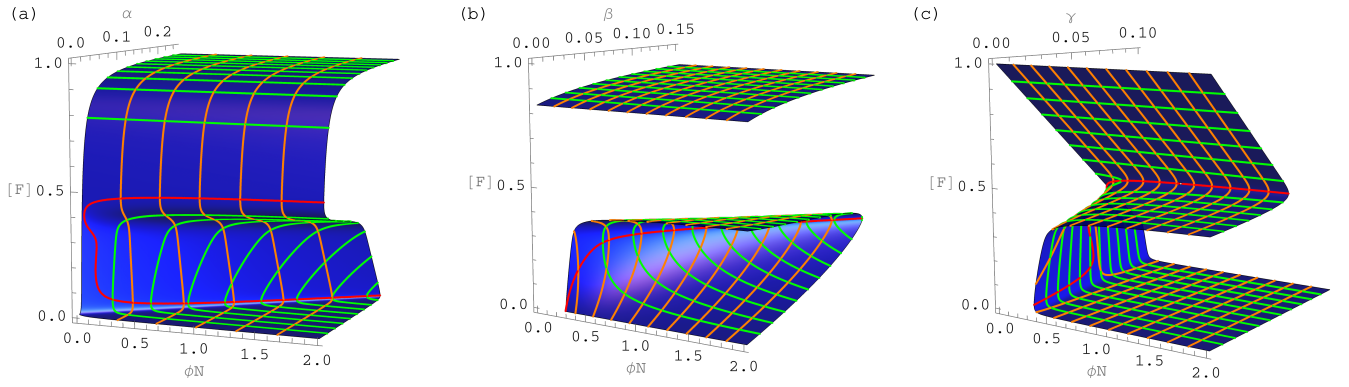

Fig. S8. Steady states and bifurcations of forest cover [F] in the two-timescale mean field of the FGBA process via Eq. S18 (blue: steady state manifold, green/orange contours at fixed axis values, red: saddle-node bifurcations) as a function of (scaled) fire ignition rate ϕN and: (a) forest spreading rate α, (b) spontaneous forest growth rate β, (c) spontaneous forest mortality rate γ. Parameters others than the ones on the axes are the same as those chosen in simulations – see Table 1.

such that the loss function is

$$\Delta^{\mathrm{loss}}_{\mathrm{F,pu}} = \phi N[\mathrm{G}]\Delta[\mathrm{F}]_{\mathrm{pu}} = \phi N p_{\mathrm{f}}[\mathrm{G}]\langle[\mathrm{FG}]\rangle^{\mathrm{u}}_{\mathrm{cg}}, \quad \text{[S17]}$$

where subscript pu refers to percolation on a square lattice with uniform random placement of forest. The final mean-field model is

$$\begin{aligned}\frac{\mathrm{d}}{\mathrm{d}t}[\mathrm{F}] &= \Delta^{\mathrm{gain}}_{\mathrm{F,mf}} - \Delta^{\mathrm{loss}}_{\mathrm{F,pu}},\\ &= \beta(1-[\mathrm{F}]) + 4\alpha[\mathrm{F}](1-[\mathrm{F}]) - \gamma[\mathrm{F}] - \phi N p_{\mathrm{f}}(1-[\mathrm{F}])\langle[\mathrm{FG}]\rangle^{\mathrm{u}}_{\mathrm{cg}}([\mathrm{F}]),\end{aligned} \quad \text{[S18]}$$

where we made explicit that $\langle[\mathrm{FG}]\rangle^{\mathrm{u}}_{\mathrm{cg}}$ is a function of [F]. This mean-field model shows clear bistability for the same parameter ranges as in simulations (Fig. S8). For the exact same parameters, this mean-field is qualitatively most comparable to simulations, but the saddle is much flatter (Fig. S9, solid blue line versus black dots).

2. Using *percolation theory*. Alternatively, we can estimate forest loss using mean burning probabilities from percolation due to a single fire. The forest perimeter exposed to fire for a given realisation is the interface of burnt grass with forest at the end of the fire $[\mathrm{FA}]^*$. When we take the expectation (for given total grass cover) and forest cells are assumed to be uniformly randomly placed, we have

$$\langle[\mathrm{FA}]^*\rangle = \langle 4[\mathrm{F}][\mathrm{A}]^*\rangle = 4[\mathrm{F}][\mathrm{G}]\langle Q_{p_{\mathrm{g}}}\rangle, \quad \text{[S19]}$$

where $\langle Q_{p_{\mathrm{g}}}\rangle$ is the mean proportion of grass that burns for given p_{g} and [G] (see Eq. S4; shown in Fig. S5b). The loss per fire in this case is then

$$\Delta[\mathrm{F}]_{\mathrm{pu}} = 4p_{\mathrm{f}}[\mathrm{F}][\mathrm{G}]\langle Q_{p_{\mathrm{g}}}\rangle, \quad \text{[S20]}$$

such that the loss function is (multiplying by $\phi N[\mathrm{G}]$)

$$\Delta^{\mathrm{loss}}_{\mathrm{F,pu}} = 4p_{\mathrm{f}}\phi N[\mathrm{G}]^2[\mathrm{F}]\langle Q_{p_{\mathrm{g}}}\rangle. \quad \text{[S21]}$$

The final mean-field model is then

$$\frac{\mathrm{d}}{\mathrm{d}t}[\mathrm{F}] = \beta(1-[\mathrm{F}]) + 4\alpha[\mathrm{F}](1-[\mathrm{F}]) - \gamma[\mathrm{F}] - 4p_{\mathrm{f}}\phi N(1-[\mathrm{F}])^2[\mathrm{F}]\langle Q_{p_{\mathrm{g}}}\rangle. \quad \text{[S22]}$$

This mean-field model has very similar steady states as Eq. S18 but the saddle is slightly lower (dotted blue line in Fig. S9). In the limit of large domain size, $\langle Q_{p_{\mathrm{g}}}\rangle$ may be replaced by the percolation probability P_∞, which is defined as the probability that a grass cell belongs to the giant component (13) (see also Fig. S11a).

Both of the estimates above assume that $p_{\mathrm{f}} = 0$ for fire spread and that p_{f} is small for loss of (uniformly randomly placed) forest due to fire. The first further assumes that fire spreads perfectly on grass ($p_{\mathrm{g}} = 1$). Therefore the two estimates are equivalent when the spreading process is pure site percolation, for which $\langle[\mathrm{FG}]\rangle_{\mathrm{cg}} = 4\langle Q_1\rangle[\mathrm{F}][\mathrm{G}]$ (equating Eq. S17 and Eq. S21), which is confirmed by Fig. S7b ('×' and '+' symbols). Comparing the estimates to recorded forest loss in fire simulations where only the assumption of random placement is taken ('∘' in Fig. S7b), one sees that the second estimate ('·' in Fig. S7b) is more accurate than the first estimate ('×' in Fig. S7b), despite that it carries more assumptions. Hence, the error due to the assumption of grass perfectly spreading compensates the error by the assumption of forest perfectly blocking fire. We expect that the difference between the two approaches will be smaller for landscapes with spatial aggregation of forest, where fires spread in pockets of high grass cover, for which $\langle Q_1\rangle - \langle Q_{0.9}\rangle$ is smaller (Fig. S7a).

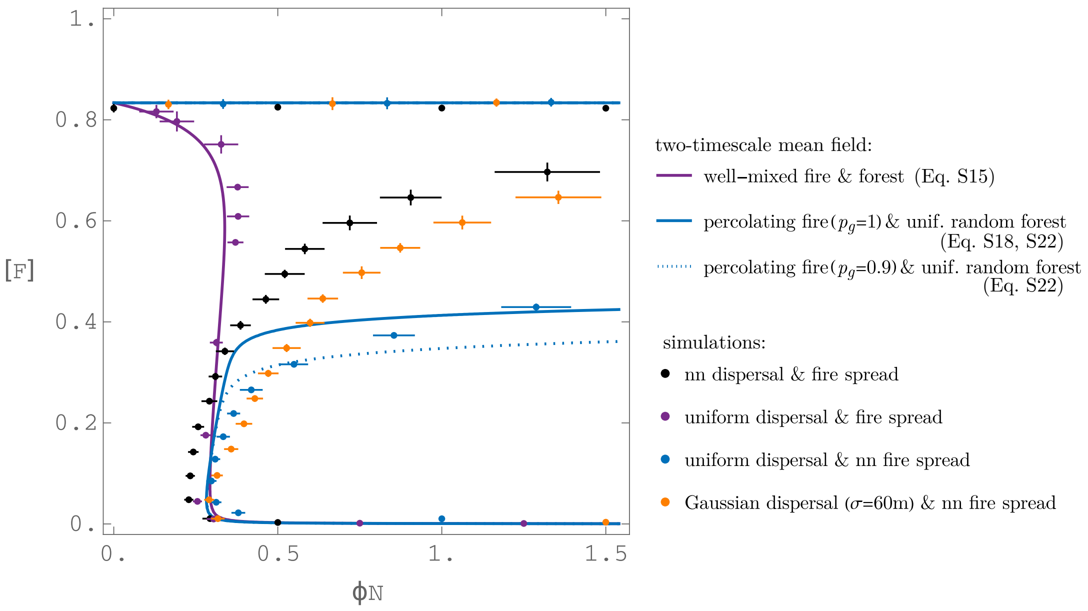

Fig. S9. Comparison of steady state forest as a function of ignition rate in the time-separated mean-field and in simulations (dots with error bars: simulations, lines: mean field). See legend for details. Note, 'uniform random' refers to the placement of forest cells in the domain.

C. Detailed comparison against simulations. Here, we compare the time-separated mean-field models to the simulations of the main text and also to other simulations with different spreading ranges for forest and/or fire. In Fig. S9, dots with error bars are simulations and lines are mean-field approximations. The black dots are the simulations from the main text, with nearest-neighbour spreading for both fire and forest. Purple dots are simulations where both fire and forest can spread to any other cell. Blue dots are simulations where forest can spread to any other cell, but fire spreads along nearest neighbours. And finally, orange dots denote simulations where forest spread occurs in a Gaussian neighbourhood with standard deviation 60m (two cells).

All simulations and approximations agree fairly well on the parameter value where the lower saddle-node bifurcation occurs. All except the fully well-mixed case agree on the stable steady states. The disagreement occurs particularly for the unstable steady states, where the effect of spatial structure is hence most pronounced and process-dependent. There is little or no bistability in the well-mixed two-timescale mean-field (purple line) but its good agreement with uniformly mixed simulations (purple dots) further indicates the validity of the assumption of time-scale separation with well-separated fire events. The mean field where fire is a site percolation process on landscapes with uniform random placement of forest (blue line), is qualitatively more correct but it has a much flatter saddle than the simulations (black dots). Hence, even the mean-field model that takes into account the effects of percolation while keeping forest cells spatially uncorrelated remains strongly biased due to the importance of spatial aggregation of forest cells. Taking larger neighbourhood sizes in the simulations does not change this (orange dots). Even compared to simulations with uniform forest dispersal (blue dots), the mean field with site percolation shows some bias, indicating that the fire spreading alone already induces some spatial structure. This is most likely caused by lower survival rates of solitary compared to aggregated forest cells.

The bias of the mean field is even more apparent in the dynamics. Figure S2 shows the same figure as Fig. 4, but with the corresponding values of the mean field with site percolation on top. Panels a–c show large differences between [FG] and $\langle[\mathrm{FG}]\rangle_{\mathrm{cg}}$ of simulations (scattered dots) versus those from the mean field (curves). In particular, while for the mean field, the perimeter is the parabola $[\mathrm{FG}] = 4[\mathrm{F}][\mathrm{G}] = 4[\mathrm{F}](1 - [\mathrm{F}])$, the perimeter of simulations lies below this parabola for any [F]. That the simulated perimeter is lower for given forest area means that forest is more spatially aggregated in simulations. This results in lower forest growth rate at any cover value (panels d–f) because fewer forest cells can expand into grass. Fire-induced damage is lower below $[\mathrm{F}] \approx 0.4$ and higher above (panels d–f). This is so because damage per fire (at given cover) is determined by two effects: exposure of forest and clustering of grass. Below $[\mathrm{F}] \approx 0.4$, there is no clustering, such that only decreased exposure due to aggregation can decrease forest loss. Above $[\mathrm{F}] \approx 0.4$, aggregation decreases clustering, such that grassland stays fully connected at higher forest cover than in the case with uniform random placement, with larger fires as a consequence. This further leads to an upward shift of the unstable forest state compared to the mean field (panels (g–i), see also Fig. S9). The effect of forest aggregation on fire spread has an equivalent in disease spread: in the SIR process, aggregation of immune individuals lowers the epidemic threshold, such that it elevates the population immunisation threshold to eradicate the epidemic (14). Note though, that, as argued above, the equivalent epidemic process to tropical fire spread in forest-grassland landscapes is not the regular SIR process, but one that has a mix of two populations: susceptibles (grass) and imperfectly immunised individuals (forest).

S5. Evolution of fronts — heterogeneous states

Here, we illustrate the case where grass and forest are initially separated into two contiguous areas with their interface extending along a straight line. Because for this type of initial conditions, the single-cluster approximation (Eq. 11) is valid, we can

focus on the evolution of the interface. As spontaneous conversion between forest and grass (with rates β and γ) increases independence between cells and promotes homogeneity at large scale, we expect the effects of heterogeneous initial conditions to be most persistent when the spontaneous conversion rates β and γ are small. Therefore, we will set $\beta = \gamma = 0$, for which Eq. 9 becomes

$$\frac{\mathrm{d[F]}}{\mathrm{d}t} = (\alpha - \phi p_{\mathrm{f}} N[\mathrm{G}])[\mathrm{FG}]. \qquad \text{[S23]}$$

Hence, the precise shape of the interface [FG] does not affect the location of the steady states, only the rate at which they are approached or receded from. The trivial steady states of Eq. S23 are $[\mathrm{F}] = 0$ and $[\mathrm{F}] = 1$ (where $[\mathrm{FG}] = 0$), which are stable, and between them, there is the saddle

$$[\mathrm{F}]^* = 1 - \frac{\alpha}{\phi N p_{\mathrm{f}}}. \qquad \text{[S24]}$$

As seen in Fig. S12, this analytical prediction (solid black) matches the controlled simulations with $p_{\mathrm{g}} = 0.9999$ (shaded blue). For $p_{\mathrm{g}} = 0.9$ (shaded red), which we used before, there is a small bias. In the limit of $N \to \infty$, Eq. S24 converges to $[\mathrm{F}]^*_\infty = 1$, implying that in an infinite domain, any positive fire rate leads to extinction of forest below $[\mathrm{F}]^* = 1$. When ignoring the effect of ash, this would also occur for heterogeneous initial conditions. That is, considering an infinite domain with many grass clusters of which the size is a random variable (with support $[0, \infty)$), there will be initial grass clusters of arbitrarily large size, which will expand and eventually drive forest extinct. However, such determinism does not occur in the simulations because at high fire rates, patches with ash start to block fires, and the rate of exposure of the forest interface to fire becomes limited by the rate at which ash is converted back into grass. As a first correction for this, one can multiply p_{f} with the average proportion of grass sites that are in the ash state after the expected waiting time between fires $1 - \exp(-\lambda/\phi N)$, such that $[\mathrm{F}]^*_\infty = 1 - \alpha/\lambda p_{\mathrm{f}}$. Keeping in mind that we are focusing on heterogeneous states, the analysis here implies that for $\gamma = \beta = 0$, there is a critical patch size above which the forest patch expands and below which it contracts. The intuition is that above the critical forest patch size, there is not enough grass area to reach the minimum number of ignitions required to erode the forest.

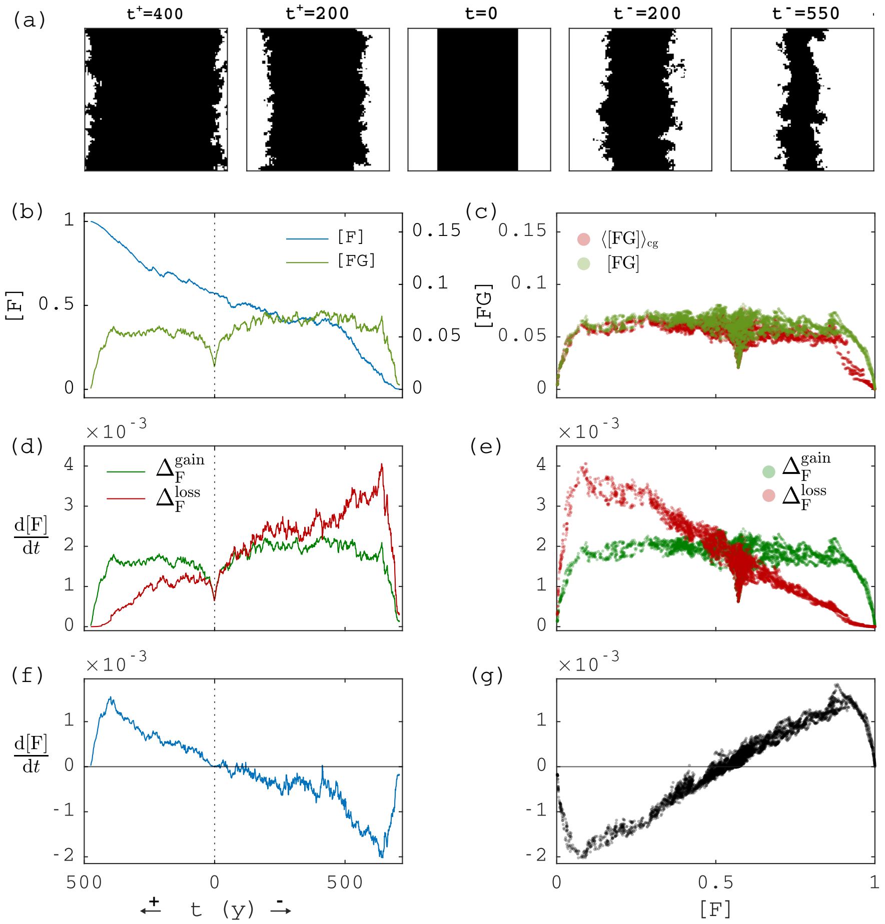

Fig. S10. Perimeter quantities and change rates according to Eq. 9 when initial conditions are heterogeneous and there are no spontaneous transitions (β=γ=0) for ϕ=$6.98 \cdot 10^{-5}$, displayed as in Figs. 3 and 4. (a) Snapshots of the domain at indicated times. (b,d,f) Cover, interface and rate of change from Eq. 9 versus time. (c,e,g) Interface, grass-cluster weighted average of the interface, and rate of change from Eq. 9 versus cover. Remaining parameters are α=0.03, ρ_{g}=10^{10}, ρ_{f}=$1.11 \cdot 10^5$, μ=10^6, λ=5.

In Fig. S10, we show how the dynamics and steady states arise from [FG], as we did in Fig. 3 and Fig. 4, but now starting with separated patches of grass and forest that interface on a line on both sides (showing for $[\mathrm{F}]_0 = 0.57$). From $[\mathrm{FG}](0) = 2L/N = 2N^{-1/2} = 0.02$, the interface quickly gains roughness due to the dynamics, until it reaches a steady state of $[\mathrm{FG}]^* \approx 6L/N = 6N^{-1/2} = 0.06$ (see Fig. S10a–c). Figure S10b confirms that $\langle[\mathrm{FG}]\rangle_{\mathrm{cg}} \approx [\mathrm{FG}]$ (except when forest cover approaches $[\mathrm{F}] = 0$ or $[\mathrm{F}] = 1$), confirming that the single-cluster approximation is valid. Away from $[\mathrm{F}] = 0$ and $[\mathrm{F}] = 1$, [FG]

stays about constant as the forest interface moves (Fig. S10b–c). Therefore the gain and loss terms (as defined in Eqs. 5 and 8) are now, respectively, constant and linearly decreasing with [F] (Fig. S10e), such that d[F]/dt increases linearly with [F] (Fig. S10g), except near [F] = 0 and [F] = 1, where it connects to 0, as here [FG] = 0.

S6. A note on finite sizes and bistability

In simple bistable chemical systems, it is known that bistability converges to an abrupt phase transition in the thermodynamic limit ($N\to\infty$) (15, 16), at a value known as the Maxwell point (e.g. 17), making the macroscopic state of the system deterministically dependent on the parameters (e.g. pressure or temperature). With only forest and grass or provided that savanna and forest components are sufficiently decoupled, the same behaviour occurs in spatial mean-field models of tropical forest, with a front between forest and nonforest that is depends deterministically on environmental drivers (18). We do not expect such determinism as $N\to\infty$ to arise in the FGBA cellular automaton. The infinite FGBA cellular automaton possesses grass clusters of arbitrary size, such that, even when assuming that fire spreads instantly on grass, there will always be some parts of the forest perimeter shielded from intruding fires by adjacent ash cells. Were it not for this shielding effect, then there would be a deterministic dependence of the dynamics on fire ignition rate away from the absorbing states: ϕ=0 would lead to forest spread while ϕ>0 would lead to forest extinction (see Section S5, for β=γ=0). In reality, finite fire spreading rates and, in particular, the effects of heterogeneity in space or time impose stronger limits on the reach of fires.

In finite domains, both the cellular automaton and scalar reaction-diffusion equations (with bistable reaction term) show bistability due to critical patch sizes or domain shapes, and dependence on interfacial characteristics (e.g. 19–21), but this correspondence requires further scrutiny. In realistic scenarios, we then suspect that the amount of bistability depends (besides the parameters) on the ratio of the characteristic interaction scale and the scale of observation. The range of interaction in turn depends on e.g. fire spreading and/or plant dispersal ranges. E.g., if we take as interaction scale the typical size of a savanna fire (assuming that it exists) $\mathcal{O}(10^{1\cdots2}\text{km}^2)$ (22), this corresponds to an area in the cellular automaton of 100×100 to 300×300 cells. This also corresponds to our observation scale (domain size) in the main text. Hence, it may be that the bistability observed in our work is a finite-size effect, and that a larger observation scale leads to more gradual transitions due to existence of multiple stable patterns (23, 24).

S7. How to include fire parameters in existing mean-field models

Previously derived mean-field models of tropical tree cover bistability did not include parameters that relate to fire. Here we give suggestions on how to include fire ignition rate and the appropriate percolation quantities, focusing on the Staver-Levin mean-field model (10, 11) of tropical savanna and/or forest bistability. We assume the reader is familiar with (10, 11).

By running an infection process on clusters obtained by standard site percolation, (11) used a mixed site-uncorrelated bond-correlated percolation process for fire (although not using this terminology). The site percolation is due to uniformly randomly distributed tree cells perfectly blocking fires and the bond percolation due to flammable cells (grass and savanna saplings in (11)) spreading fires with a given probability. The correlation in bond occupation probability occurs due to the infection dynamics, as explained in Section S2C. One can use the complement of the burning probability of this mixed percolation process, i.e. $1-\langle Q_{p_{\rm b}}(p_s)\rangle$, as survival probability instead of that used in (11) (see Fig. S11b for a plot of $\langle Q_{p_{\rm b}}(p_s)\rangle$ as a function of the infection probability between flammable cells p_b and the probability of a cell being flammable p_s). If one does so, one can write the mean-field recruitment rate of savanna saplings during a small time interval as $\omega(p_b,p_s)$[S], where ω is

$$\omega(p_b,p_s) := \max[\omega_0 - \phi N p_s \langle Q_{p_{\rm b}}(p_s)\rangle \Delta\omega, 0], \qquad \text{[S25]}$$

with $\Delta\omega$>0 the per fire decrease in recruitment rate due to burning and ϕ the fire ignition rate in flammable cells. Note that, according to (11), the total flammable area is p_s = [S] + [G] (i.e., the area of grass and savanna saplings). The reasoning is that there are on average $\phi N p_s$ ignitions, each causing a fire that on average affects a proportion $\langle Q_{p_{\rm b}}(p_s)\rangle$ of flammable cells, thereby lowering the recruitment rate by an amount $\Delta\omega$. If [S] and [T] cells are uniformly randomly placed in the area affected by fire, then it follows that the recruitment rate is $\omega(p_b,p_s)$[S].

For the approximate effect on forest trees (as included in 10), one needs to take into account that forest trees are assumed (in 10) to block fires perfectly. Therefore, they are not in the flammable part of the landscape, but instead share an interface with it. The mean-field rate of forest loss due to fire during a small time interval is then $\zeta(p_b,p_s)$[F], where ζ is

$$\zeta(p_b,p_s) := 4\phi N p_s^2 p_{\rm f} \langle Q_{p_{\rm b}}(p_s)\rangle, \qquad \text{[S26]}$$

with p_s = [S] + [G] also. The reasoning here is as follows. There are on average $\phi N p_s$ ignitions, each causing a fire that affects a proportion $\langle Q_{p_{\rm b}}(p_s)\rangle$ of the landscape. Assuming that occurrences of burnt and forest cells are uncorrelated, one can write the interface between them as the number of forest-burnt pairs in a lattice: $4(\langle Q_{p_{\rm b}}(p_s)\rangle p_s)$[F]. For each such pair, there is a probability $p_{\rm f}$ of spreading into forest, such that (when using the approximation of small $p_{\rm f}$ as in the main text), the resulting forest loss is $4\phi N p_s^2 p_{\rm f}\langle Q_{p_{\rm b}}(p_s)\rangle$[F].

For large domains, one may replace $\langle Q_{p_{\rm b}}(p_s)\rangle$ by the percolation probability $P_\infty(p_b,p_s)$ (shown in Fig. S11a), which is the probability that a flammable cell is part of the giant connected component.

References

1. T Tomé, MJ De Oliveira, *Stochastic dynamics and irreversibility.* (Springer), p. 394 (2015).
2. IZ Kiss, JC Miller, PL Simon, *Mathematics of epidemics on networks: From exact to approximate models*, Interdisciplinary Applied Mathematics. (Springer International Publishing, Cham) Vol. 46, pp. 1–413 (2017).
3. R Durrett, Spatial epidemic models in *Epidemic models : their structure and relation to data.* (New York, NY), pp. 187–201 (1995).
4. L Hébert-Dufresne, et al., Edge fires drive the shape and stability of tropical forests. *Ecol. Lett.* **21**, 794–803 (2018).
5. T Tomé, RM Ziff, Critical behavior of the susceptible-infected-recovered model on a square lattice. *Phys. Rev. E - Stat. Nonlinear, Soft Matter Phys.* **82**, 051921 (2010).
6. YY Tarasevich, SC Van Der Marck, An investigation of site-bond percolation on many lattices. *Int. J. Mod. Phys. C* **10**, 1193–1204 (1999).
7. F Crameri, Scientific colour maps (2018) http://doi.org/10.5281/zenodo.1243862.
8. L Hébert-Dufresne, A Allard, Smeared phase transitions in percolation on real complex networks. *Phys. Rev. Res.* **1**, 13009 (2019).
9. D Stauffer, A Aharony, *Introduction to percolation theory.* (CRC press), (1994).
10. AC Staver, SA Levin, Integrating theoretical climate and fire effects on savanna and forest systems. *The Am. Nat.* **180**, 211–224 (2012).
11. E Schertzer, AC Staver, SA Levin, Implications of the spatial dynamics of fire spread for the bistability of savanna and forest. *J. Math. Biol.* **70**, 329–341 (2014).
12. DD Patterson, SA Levin, C Staver, JD Touboul, Probabilistic Foundations of Spatial Mean-Field Models in Ecology and Applications. *SIAM J. on Appl. Dyn. Syst.* **19**, 2682–2719 (2021).
13. K Christensen, NR Moloney, *Complexity and Criticality*, Imperial College Press Advanced Physics Texts. (Imperial College Press and Distributed) Vol. 1, (2005).
14. MJ Keeling, The effects of local spatial structure on epidemiological invasions. *Proc. Royal Soc. B: Biol. Sci.* **266**, 859–867 (1999).
15. H Ge, H Qian, Thermodynamic limit of a nonequilibrium steady state: Maxwell-type construction for a bistable biochemical system. *Phys. Rev. Lett.* **103**, 148103 (2009).
16. U Thiele, T Frohoff-Hülsmann, S Engelnkemper, E Knobloch, AJ Archer, First order phase transitions and the thermodynamic limit. *New J. Phys.* **21**, 123021 (2019).
17. N Goldenfeld, *Lectures on phase transitions and the renormalization group.* (CRC Press), pp. 1–394 (2018).
18. B Wuyts, AR Champneys, N Verschueren, JI House, Tropical tree cover in a heterogeneous environment: a reaction-diffusion model. *PLoS ONE* **14**, e0218151 (2019).
19. A Liehr, *Dissipative solitons in reaction diffusion systems.* (Springer) Vol. 70, (2013).
20. N Goel, V Guttal, SA Levin, AC Staver, Dispersal increases the resilience of tropical Savanna and forest distributions. *Am. Nat.* **195**, 833–850 (2020).
21. SA Levin, Non-uniform stable solutions to reaction-diffusion equations: Applications to ecological pattern formation in *Pattern Formation by Dynamic Systems and Pattern Recognition*, Springer Series in Synergetics, ed. H Haken. (Springer-Verlag) Vol. 5, pp. 210–222 (1979).
22. N Andela, et al., The global fire atlas of individual fire size, duration, speed and direction. *Earth Syst. Sci. Data* **11**, 529–552 (2019).
23. M Rietkerk, et al., Evasion of tipping in complex systems through spatial pattern formation. *Science* **374** (2021).
24. R Bastiaansen, HA Dijkstra, ASVD Heydt, Fragmented tipping in a spatially heterogeneous world. *Environ. Res. Lett.* **17**, 045006 (2022).

S8. Additional figures

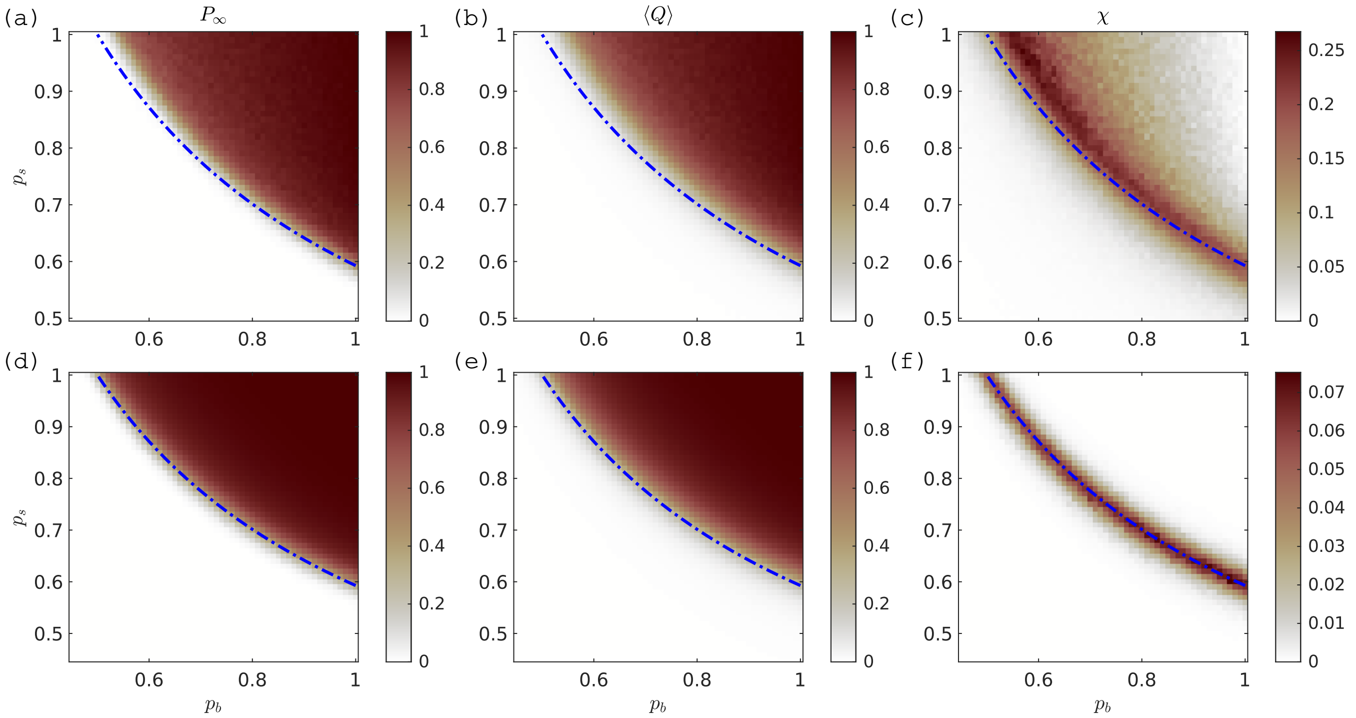

Fig. S11. GBA process with ϕ=λ=0 (equivalent to SIR spreading on the square lattice) in terms of bond occupation probability $p_b := p_{\rm g}$ (Eq. 3) and site occupation probability $p_s := [{\rm G}]_0$ (a-c) and compared to standard mixed site-bond percolation (d-f). Shown quantities as a function of bond and site occupation probability (based on 1024 realisations for (a-c) and on 512 realisations for (d-f)): (a,d) percolation probability P_∞, (b,e) mean proportion of burnt grass cells $\langle Q\rangle$ (see Eq. S4), and, (c,f) susceptibility χ=$\langle Q^2\rangle/\langle Q\rangle$. Percolation probability is defined as the probability that any grass cell belongs to the giant component (13). Susceptibility is defined here as in (8), using Q as order parameter. The dash-dotted blue line indicates the location of the infinite-size percolation threshold for uncorrelated mixed site-bond percolation (taken from (6)). For a domain size of 100x100 cells and at the shown resolution, the percolation threshold of mixed site-bond percolation matches that of the infinite size system (f). The GBA process' percolation threshold lies at higher values (c) due to spatial correlation of $p_{\rm g}$ as explained in the text. The top row is more noisy than the bottom row because for standard mixed percolation, we were able to obtain the whole distribution of cluster sizes for a each realisation and computed their statistics using percolation theory (13), whereas for the GBA process, each simulation only resulted in one sample. The colour scale was taken from (7).

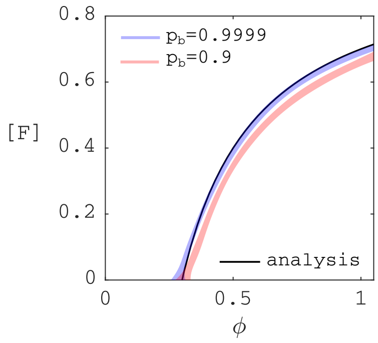

Fig. S12. Saddle of Eq. S23 via Eq. S24 (single cluster – solid black) compared to controlled simulations (shaded red: $p_{\rm g} = \rho_{\rm g}/(\rho_{\rm g} + \mu) = 0.9$, shaded blue: $p_{\rm g} = 0.9999$) in case of heterogeneous initial conditions and without spontaneous interactions (β=γ=0). Other parameters: α=0.03, $\rho_{\rm f}$=1.11 $\cdot$ 10^5, μ=10^6, λ=5.
